# Influence of heterotrophs on phage infection of marine picocyanobacteria

**DOI:** 10.1128/msystems.00769-26

**Published:** 2026-07-20

**Authors:** Allison Coe, Sierra M. Parker, Nhi N. Vo, Sean M. Kearney, Shaul Pollak, Konnor von Emster, James I. Mullet, Kurt G. Castro, Sallie W. Chisholm

**Affiliations:** 1 Department of Civil and Environmental Engineering, Massachusetts Institute of Technology 2167, Cambridge, Massachusetts, USA; 2 Centre for Microbiology and Environmental Systems Science, University of Vienna 27258, Vienna, Austria; 3 Department of Biology, Massachusetts Institute of Technology 2167, Cambridge, Massachusetts, USA; Institute for Systems Biology, Seattle, Washington, USA

**Keywords:** cyanophages, picocyanobacteria, cyanobacteria, heterotrophic bacteria, cell-cell interaction, metabolite exchange, microbial ecology, organic carbon, infection, microbial communities

## Abstract

Picocyanobacteria *Prochlorococcus* and *Synechococcus* coexist with lytic phages and heterotrophic bacteria in the oceans. These phages are a significant cause of mortality, while heterotrophic bacteria can increase *Prochlorococcus* fitness by reducing oxidative stress and cross-feeding during extended darkness. Studies of *Prochlorococcus-*phage interactions are often done with xenic cultures, as it has been historically difficult to obtain and maintain heterotroph-free cultures. Here, we examine how heterotrophic bacteria affect phage infection dynamics in *Prochlorococcus* and *Synechococcus* by comparing infections in cultures with and without heterotrophs. We found that *Prochlorococcus* populations resumed growth following infection only when heterotrophs were present, independent of phage-host or heterotroph-host ratios. In *Synechococcus* phage-host pairings, outcomes varied, suggesting that heterotrophic effects depend on the phage-host interaction. When hosts recovered from infection, heterotrophs appeared to facilitate this by mitigating oxidative stress and possibly supplying organic carbon sources that support post-infection growth. Recovered *Prochlorococcus* and *Synechococcus* populations were resistant to reinfection when transferred to fresh media. However, comparisons of host sequences before and after infection argue against genetic change as the mechanism of tolerance. Instead, we infer that hosts undergo non-genetic adaptations during recovery in the presence of heterotrophs, likely driven by heterotroph-derived organic compounds that reshape host metabolism and confer protection against future lysis. During this process, heterotrophic community composition shifted dynamically across sequential transfers, yet host recovery remained consistent, suggesting that either community function or host adaptations maintained support for growth.

**IMPORTANCE:**

This study uncovers a previously unrecognized role of heterotrophic bacteria in shaping phage infection outcomes in marine cyanobacteria. Our findings demonstrate that the presence of heterotrophs can enable *Prochlorococcus* to recover from phage infection, irrespective of phage:host or heterotroph:host ratios. In contrast, *Synechococcus* exhibited variable outcomes, suggesting that the impact of heterotrophs is dependent on the heterotroph-host pairing. Recovery after phage infection appears to be driven by heterotroph-facilitated mitigation of oxidative stress and provision of organic carbon, which may alter host metabolism and contribute to protection against future phage infection. These results highlight the importance of the microbial community, particularly heterotrophs, in shaping cyanobacteria-phage dynamics and highlight the need to reframe host-phage interactions within a broader ecological framework.

## INTRODUCTION

Marine picocyanobacteria *Prochlorococcus* and *Synechococcus* are small (~<1–2 µm) primary producers in the marine community with a global population estimated at 3 × 10^27^ and 7 × 10^26^ cells, respectively (1). These genera are found in abundance (~10^4^–10^5^ cells mL^−1^) in the upper 200 m of the world’s oceans and coexist with ~10^5^–10^6^ mL^−1^ of heterotrophic bacteria and ~10^5^–10^7^ phage mL^−1^, with which they have the potential to interact (2–5). *Prochlorococcus* and *Synechococcus* display high rates of photosynthetically derived fixed carbon release (2, 6), potentially fueling up to ~40% of subtropical heterotrophic bacterial growth (7). In xenic cultures, defined here as a single cyanobacterial strain coexisting with its co-isolated native heterotrophic community, without added carbon to media, the organic carbon released by cyanobacteria can support a wide diversity of heterotrophic species (8, 9).

The presence of heterotrophs in a culture also influences the metabolism of *Prochlorococcus*. Indeed, within 6 h of introducing a heterotroph to the *Prochlorococcus* culture, the cells’ transcriptional profile shifts in ways that implicate a number of cellular activities and metabolic pathways, such as stress responses, photosynthesis, central carbon metabolism, amino-acid biosynthesis, and membrane transport (10). While the organic compounds produced by *Prochlorococcus* support heterotrophic growth, the heterotrophs, in turn, recycle components of the dissolved organic matter (DOM) and release metabolites that *Prochlorococcus* can utilize—creating a dynamic, mutualistic exchange cycle within the community (10–12). This is further supported by *Prochlorococcus*’s demonstrated ability to transition to mixotrophic metabolism, facilitating heterotrophic assimilation of various compounds, including amino acids and glucose (13–16). In addition, certain “helper” heterotrophs mitigate oxidative stress in *Prochlorococcus*—an environmental challenge that *Prochlorococcus* is particularly sensitive to as it does not produce catalase (17–19).

*Prochlorococcus* and *Synechococcus* are vulnerable to infection by cyanophages, which outnumber cyanobacteria by twofold to fourfold in the North Pacific, but infections are estimated to contribute only up to 5% of *Prochlorococcus*’s daily mortality, suggesting successful encounters and infections are rare (20). On a global scale, lysed prokaryotic cells contribute ~145 Gt C per year to the DOM pool in tropical and subtropical oceans (21), representing a major flux within the carbon cycle. In the case of cyanobacteria, phage-induced lysis fuels heterotrophic growth, driving shifts in the community structure (22, 23). Notably, DOM produced by viral lysis of *Prochlorococcus* is compositionally distinct and more bioavailable than what is passively released by exponentially growing *Prochlorococcus*, and its composition can vary with different phage-host combinations, leading to differences in the diversity and structure of the heterotrophic community (24). DOM produced by *Synechococcus* viral lysates has been found to be rich in nitrogen-containing compounds (22, 25), and as the lysates are metabolized by heterotrophic bacteria, inorganic nutrients, such as ammonium, are remineralized (22). As pointed out earlier, heterotrophs influence metabolic pathways within *Prochlorococcus* cells; therefore, an increase in heterotroph abundance and a change in community composition due to phage infection could further alter *Prochlorococcus*’s and *Synechococcus*’s metabolism, potentially impacting subsequent cyanophage infection dynamics.

Numerous studies have explored pairwise phage-cyanobacteria and heterotroph-cyanobacteria interactions. Here, we take the next step in understanding community dynamics by examining their three-way dynamics by assessing whether the presence of heterotrophic bacteria in clonal cultures of cyanobacteria influences phage infection dynamics—either directly or indirectly. To this end, we compare infection dynamics in axenic (cyanobacteria only), monoxenic (cyanobacteria plus a single heterotroph, *Alteromonas macleodii* MIT1002, hereafter referred to as *Alteromonas*), and xenic (cyanobacteria plus a community of heterotrophs that were originally co-isolated with them) *Prochlorococcus* and *Synechococcus* cultures. Finding that the presence of heterotrophs promotes host survival, we next examine how the ratios of phage:host and heterotroph:host impact host recovery from phage infection. We then do a series of experiments designed to help understand how heterotrophs might infer resistance to phage infection. Finally, we investigated whether shifts in the heterotrophic community following sequential phage infections might play a role in shaping host responses, including the potential development of phage resistance.

## MATERIALS AND METHODS

### Cultures

Axenic (cyanobacteria alone), monoxenic (cyanobacteria plus a single heterotroph), and xenic (cyanobacteria plus a community of heterotrophs that were originally co-isolated with them) *Prochlorococcus* (MED4) and *Synechococcus* (WH7803, WH8102, and MITS9451) cultures were grown in 0.2 μm filtered sterile natural seawater amended with Pro99 nutrients prepared as previously described (26). Strains WH7803 and WH8102 have been recently reclassified as *Parasynechococcus* (27), but we are using the traditional designation “*Synechococcus*” for consistency with prior literature and earlier studies on these strains and continuity for readers (28). Prior to addition to cyanobacteria cultures, *Alteromonas macleodii* MIT1002 cultures were grown in 0.2 μm filtered sterile natural seawater amended with ProMM nutrients as previously described (29). While we do not have direct *Alteromonas* abundance data during the experiment, given typical heterotroph growth rates in Pro99 media, including those of *Alteromonas* which have a doubling time of ~3–4 h (30, 31), indicate that a population initiated from a single cell would undergo only ~6–8 doublings within 24 h (resulting in ~10^2^–10^3^ cells mL^−1^). This growth would be insufficient to reach the highest starting inoculum cell density (~10⁶ cells mL⁻¹), and thus, differences in *Alteromonas* density between treatments are expected to persist when *Prochlorococcus* lysis begins between 24 and 48 h. All cells were grown in continuous light at ~15 µmol photons m^−2^ s^−1^ at 24°C. At the beginning of each experiment, exponentially growing cultures of the cyanobacterium were enumerated by flow cytometry (described below) so that initial concentrations of *Prochlorococcus* could be set at 1 or 1.5 × 10^7^ cells mL^−1^. In cases where *Alteromonas* was added, its initial concentration was set to be 10-fold less (1 or 1.5 × 10^6^ cells mL^−1^) than that of the cyanobacterium, unless otherwise stated. This dilution was selected on the basis that, throughout exponential growth, *Alteromonas* abundances in *Prochlorococcus* co-cultures are generally an order of magnitude lower (12, 32, 33). *Alteromonas* was chosen because the *Prochlorococcus–Alteromonas* interaction has become a well-established model system for studying cyanobacteria–heterotroph interactions (10, 12, 34, 35). It has also been employed in co-culture experiments with *Synechococcus*, including mixed cyanobacterial systems (*Prochlorococcus*, *Synechococcus*, and *Alteromonas*) (36). In xenic cultures, heterotrophs were 4–10 times less abundant than the cyanobacteria. The composition of these communities (except for those with *Synechococcus* MITS9451) was described in Kearney et al. (9). Although the composition may have slightly changed since then, we note that the current communities in xenic *Prochlorococcus* MED4 cultures generally align with those previously reported.

Cells were grown in either 96-well cell culture plates (cat#353072, Corning, Glendale, AZ) or 25 mm borosilicate test tubes (cat#47729-586, Avantor VWR, Radnor, PA) with five or three biological replicates, respectively. Growth was monitored via bulk chlorophyll fluorescence in plates (Synergy2, Agilent BioTek, Santa Clara, CA) or in tubes (TD700, Turner Designs, Sunnyvale, CA) and by flow cytometry (see below). For experiments involving reactive oxygen species (ROS) and organic carbon additions, 5 M stocks were made (5 mM final concentration in culture) with ultra-pure water and cell-culture grade chemicals (e.g., Bioxtra, Millipore Sigma, Burlington, MA). Stocks were sterilized by filtering through a 0.2 µm Supor syringe filter (cat# 4506, Cytiva, Marlborough, MA) and stored at 4°C until use.

### Phages

In this study, we used three cyanophages: the podo- and T7-like specialist phage P-SSP7 and the myo- and T4-like phage P-HM2 to infect *Prochlorococcus* MED4, and the myo- and T4-like generalist phage Syn9 to infect *Synechococcus* WH8102, WH7803, and MITS9451. Phage titers were determined using the most probable number method as described by Jarvis et al. (37), and phages were added at the onset of each experiment at a multiplicity of infection (MOI) of 0.1, unless otherwise noted. Phage and cyanobacteria were co-incubated for 60 min to allow for adsorption, after which Pro99 medium was added to bring the final well volume to 200 µL.

### Flow cytometry

*Prochlorococcus* cell abundances were measured as previously described (33) using a Guava 12HT flow cytometer (Luminex Corp., Austin, TX, USA) equipped with a 488 nm blue laser. Cells were analyzed based on chlorophyll autofluorescence (692/40 nm) and DNA content (530/40 nm) following staining with 1× SYBR Green I (Invitrogen, Grand Island, NY, USA) and a 60 min dark incubation prior to analysis. All flow cytometry data were processed and analyzed using FlowJo software (version 10.8.1; FlowJo LLC, BD Life Sciences, Ashland, OR, USA).

### DNA sequencing and heterotrophic community analysis

To examine genetic mutations and identify shifts in heterotroph composition across sequential infections, genomic DNA was extracted from biological triplicate cultures harvested at the late exponential phase following both the first and second infections. Cells from 15 mL of culture were spun down at 7,197 *× g* for 20 min with 0.003% Pluronic F-68 Polyol (MP Biomedicals, Cat# 2750049), which enhanced cell aggregation and biomass recovery. The resulting pellets were stored at −80°C prior to DNA extraction. Genomic DNA was isolated using the protocol found in Wilson et al. (38), modified for *Prochlorococcus* as described in Coe et al. (39). Sequencing libraries were prepared with the Illumina NexteraXT kit (cat# FC-131-1096, Illumina, San Diego, CA) following the manufacturer’s protocol. Samples were indexed using the TG NexteraXT Index Kit v2 Set A (cat# TG-131-2001 for kit A or ending in 2 for kit B), and sequencing was performed on the Illumina NextSeq500 at the MIT BioMicro Center. Sequencing reads were deposited to SRA under BioProject PRJNA1302459 (Table S1).

To identify single nucleotide polymorphisms (SNPs) and indels before and after sequential phage infections in monoxenic and xenic *Prochlorococcus* MED4 and xenic *Synechococcus* MITS9451 cultures, we used a modified version of the variant-calling workflow described by Conwill et al. (40) and compared this to results obtained using breseq v0.39.0 (41). Key adjustments for the Conwill workflow included setting the minimum allele frequency threshold to 0.1 and increasing the maximum read depth per position to 5,000. For the Conwill workflow, sequencing reads were trimmed with Cutadapt v.1.18 (42), aligned to reference genomes *Prochlorococcus* MED4 (IMG accession Ga0065304_11) and *Synechococcus* MITS9451 (NCBI accession NZ_CP138971) using bowtie2 v2.2.6 (43), and variants were called with Samtools v1.9 and BCFtools v.1.2 (44) (Table S2). For breseq, polymorphism mode was run on default settings, which include a minimum allele frequency of 5% (Table S3). To identify phage-specific mutations, we retained only variants present in at least two biological replicates and unique to both the first and second infections, while excluding any variant that was detected in all treatments.

To detect larger genomic additions, such as CRISPR-Cas systems, prophages, or other mobile elements, we first mapped cyanobacterial reads to a reference genome (see above) using bowtie2 v2.5.4 with default settings (43) and performed *de novo* assembly of each sample using SPAdes v4.2.0 (45). Contigs were further filtered to include only those longer than 1,000 bp, except for replicate B of MITS9451, for which contigs >500 bp were used due to limited assembly. Pairwise comparisons of the filtered contigs were performed using MUMmer4 v4.0.1 (46), comparing the no-phage control (before infection) to the state after the first infection. MUMmer plots revealed continuous diagonals with no breaks (Fig. S1), indicating no large insertions or deletions were detected.

To characterize the heterotrophic community co-isolated with the host cells that were serially transferred over many years, we used ProSynTax (39), a curated protein sequence data set for classifying metagenomic sequences, and a modified version of its workflow, available at https://github.com/nhinvo/pro-het-phage-interaction. Because of an error in recording the concentration and volume of an added DNA standard during sample prep, which was originally intended to obtain absolute quantification, we used relative quantification instead. To prevent interference from the standard, reads were processed through a two-step filtering approach, as read mapping may not detect all standard-derived reads. Reads were first trimmed using BBDuk v39.18 (47), then mapped to the *Thermus thermophilus* ATCC BAA-163 reference genome (NCBI accession AE017221) using bowtie2 v2.5.4 (43), and standard-derived reads were removed with SeqKit v2.10.0 (48). Taxonomic classification of the resulting reads was performed with Kaiju v1.10.1 (49) against the ProSynTax v1.1 data set (39), which includes an extensive reference set for heterotroph taxonomy. Any remaining reads classified as *Thermus thermophilus* were filtered to ensure accurate downstream analysis. Genera that did not reach at least 1% of classified reads in either the pre- or post-infection samples were grouped under the category “Others” (Table S3).

### Statistical analysis of heterotrophic composition changes

Changes in taxonomic composition in xenic cultures of *Prochlorococcus* MED4 and *Synechococcus* MITS9451 were analyzed using linear mixed-effects models. Relative abundance was modeled as the response variable with time point and taxonomic group (genus or class) as fixed effects, including their interaction, and replicate as a random effect. Significance of fixed effects was assessed using *F*-tests with Kenward-Roger degrees of freedom as implemented in the lmerTest v3.2-0 R package (50). *Post hoc* pairwise comparisons of estimated marginal means were performed using Tukey-adjusted contrasts, with *P* values further corrected for multiple testing using the false discovery rate method (51), as implemented in the emmeans v2.0.2 R package (52). Analyses were conducted independently at the class and genus levels for each host to assess shifts in heterotrophic community composition over time.

## RESULTS AND DISCUSSION

### Heterotrophic effects on *Prochlorococcus* phage infection

**Fig 1:**
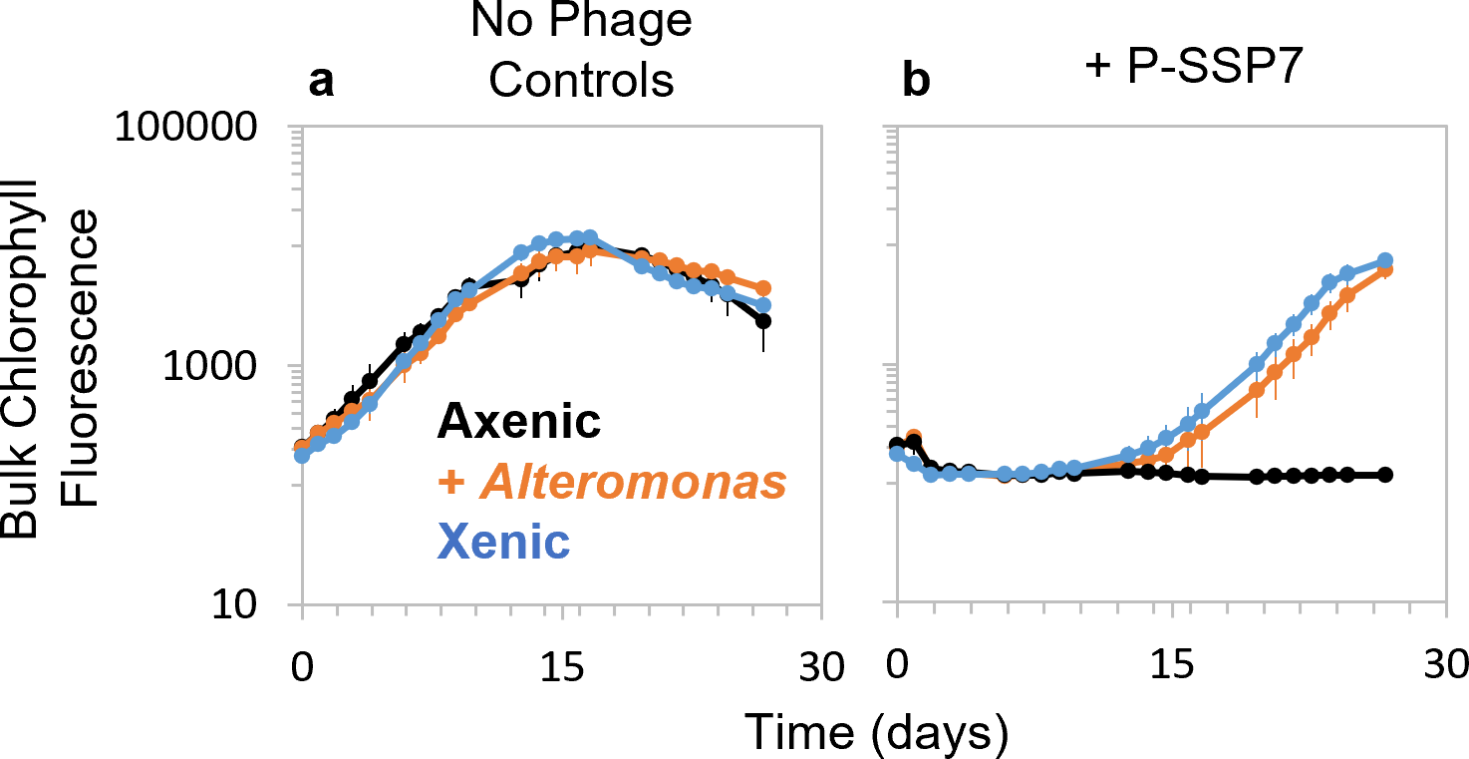
Heterotroph presence enables recovery post-phage infection of *Prochlorococcus*. Axenic (black), monoxenic (+*Alteromonas*) (orange), and xenic (community of heterotrophs) (blue) cultures of *Prochlorococcus* MED4 without (**a**) or with (**b**) cyanophage P-SSP7. Phages were added at the onset of the experiment, and the culture dynamics were monitored via bulk culture chlorophyll fluorescence. Xenic cultures were maintained separately from axenic cultures, each harboring an independently maintained heterotrophic community.

Rendering *Prochlorococcus* cultures axenic has been historically challenging due to their dependence on co-occurring heterotrophs to mitigate oxidative stress (17, 18), and consequently, early studies of phage infection were conducted on xenic cultures (53, 54). With recent advances facilitating the routine isolation of axenic cultures (29, 55), we systematically investigated the role of heterotrophs in shaping infection dynamics. We grew *Prochlorococcus* MED4 under three conditions: axenic, monoxenic (referred to as “+*Alteromonas*” in figures), and xenic (cyanobacteria plus a community of heterotrophs that were originally co-isolated with them) (9). Experimental cultures were infected with podovirus-like P-SSP7 (Fig. 1). Control cultures (“no-phage control”) grew as expected, with minimal differences across the three culture types (Fig. 1a). Following phage introduction, all treatments underwent lysis, as evidenced by the flat-lining of bulk culture chlorophyll fluorescence relative to the controls for 10 days. Only cultures with heterotrophs—both xenic and +*Alteromonas*—resumed growth after ~10 days post-infection (Fig. 1b).

**Fig 2:**
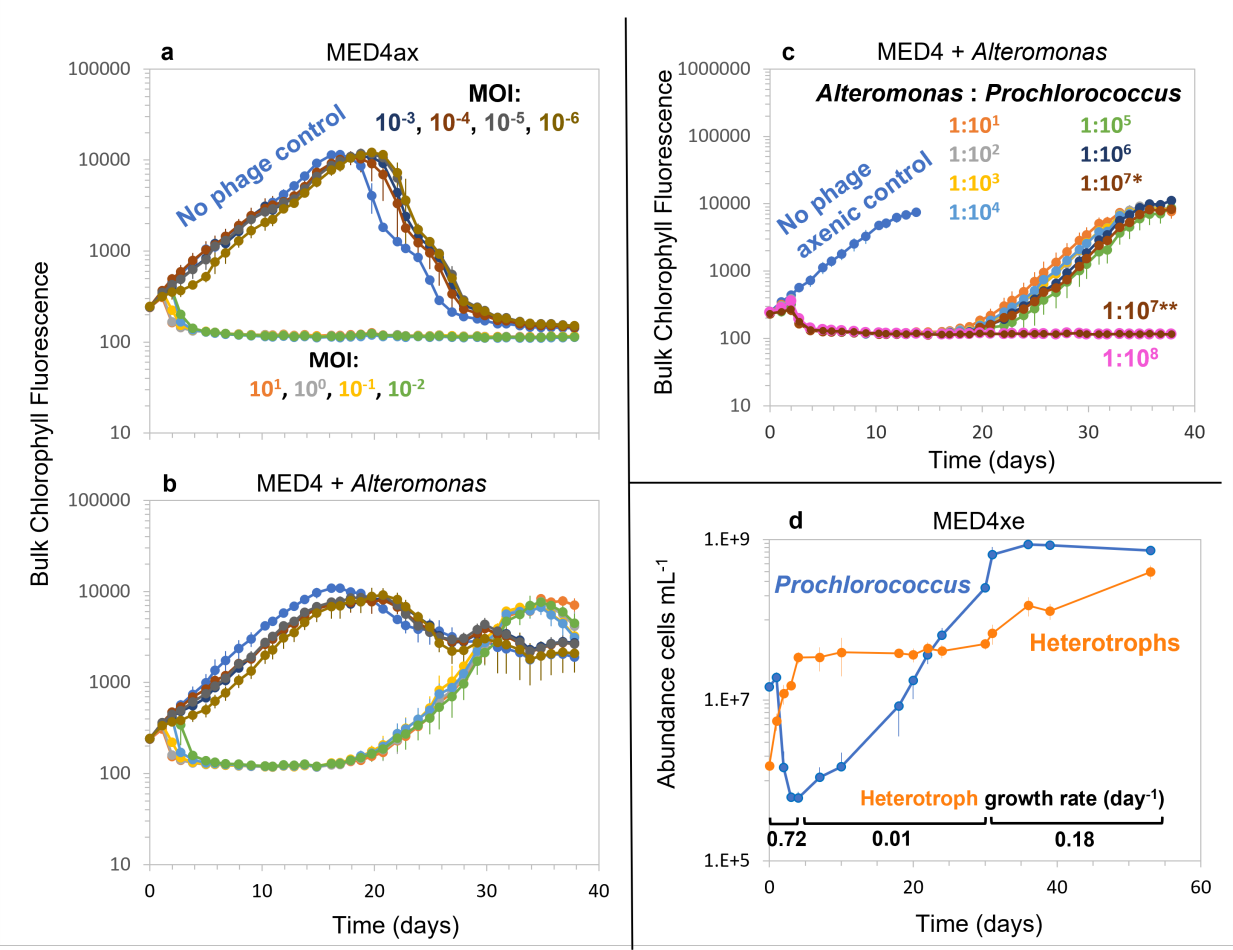
Phage infection drives *Prochlorococcus* decline and transient heterotroph increase, independent of initial phage or heterotroph ratios. (**a**) Axenic and (**b**) monoxenic (+*Alteromonas*) *Prochlorococcus* MED4 cultures infected using MOIs (phage:host ratio) ranging from 10 to 10^−6^. (**c**) Infected monoxenic *Prochlorococcus* MED4 (+*Alteromonas*) cultures grown at *Alteromonas*:*Prochlorococcus* ratios from 1:10 to 1:10^8^. In the 1:10^7^ ratio (brown), one out of the six biological replicates resumed growth (*) while the remainder did not resume growth (**). The 1:10^8^ ratio (pink), effectively zero, served as a negative control for *Alteromonas* presence. (**d**) Abundance of *Prochlorococcus* MED4 (blue) and their associated heterotrophs (orange) in xenic cultures after infection. Heterotroph growth rates (day⁻¹) are shown in black during lysis, at the onset of MED4 recovery, and once MED4 abundance exceeds heterotroph abundance. All cultures, except the no-phage control (blue) in panels **a–c**, were infected with podo-like cyanophage P-SSP7 at the onset of the experiment, and infection dynamics were monitored via chlorophyll fluorescence (**a–c**) or flow cytometry (**d**).

This difference posed the question of whether phage had adsorbed to the heterotrophs, effectively decreasing the MOI (i.e., ratio of phage to *Prochlorococcus* host), thus diluting phage encounters with *Prochlorococcus*. To explore this, we adjusted the MOI—set to 0.1 in all other experiments unless otherwise specified—to see if it would influence recovery, comparing axenic and monoxenic *Prochlorococcus* MED4 cultures (Fig. 2a and b). Across a wide MOI range (10–0.001), both axenic and monoxenic cultures showed similar infection dynamics (±2 days), while MOIs below 0.0001 failed to establish infection in either culture (Fig. 2a and b). Only the cultures containing *Alteromonas* recovered post-infection, and the timing of recovery was consistent across all effective MOIs (Fig. 2b). Notably, in the low-MOI cultures that failed to lyse, *Alteromonas* also prolonged *Prochlorococcus* survival into the post-stationary (decline) phase compared to axenic cultures, which rapidly collapsed, consistent with observations by Weissberg et al. (35), who suggested that these two strains exhibit mutualistic interactions. Thus, recovery only occurred in the presence of heterotrophs and was independent of the initial phage-to-host ratio, suggesting that it was driven by heterotroph-associated processes rather than by the magnitude of the initial infection.

As the timing of recovery is decoupled from the host:phage ratio, we wondered whether the concentration of heterotrophs at the onset of the experiment would have an influence on these dynamics. What is the minimum concentration of heterotrophs to facilitate recovery after infection? To test this, we grew axenic *Prochlorococcus* MED4 with varying initial ratios of *Alteromonas*:*Prochlorococcus* cells, ranging from 1:10 to 1:10^8^. The actual starting *Prochlorococcus* abundance was 1.5 × 10^7^, while *Alteromonas* concentrations ranged from 1.5 × 10^6^ to 1.5 × 10^−1^ cells mL^−1^, with the higher concentration representing the standard 10-fold dilution used in all experiments and the lowest serving as the control for the absence of *Alteromonas*. Cultures were then infected with P-SSP7 (Fig. 2c). *Prochlorococcus* resumed growth at similar times across all treatments where heterotrophs were present, regardless of the initial *Alteromonas*:*Prochlorococcus* ratio (Fig. 2c, days 17–18). The only exception was the 1:10^7^ ratio, where only one out of the six replicates resumed growth (Fig. 2c, brown line). This anomaly could be attributed to the inherent errors that come with pipetting samples of cells with such low abundance. Overall, these results show that as long as heterotrophs are present—even at one cell per 10,000,000 *Prochlorococcus* cells—*Prochlorococcus* can recover post-infection. Thus, the heterotrophs are either important for phage resistance or recovery post-infection, irrespective of MOI or heterotroph abundance.

### Heterotroph abundance during phage infection

When heterotrophs are introduced to *Prochlorococcus* cultures maintained in natural seawater-based media without added organic compounds, they can grow initially, fueled by background seawater organic matter. Once this is depleted, however, heterotroph growth is entirely dependent on photosynthate released by *Prochlorococcus*, limiting their proliferation (12, 30, 32). Evidence also shows that phage-induced lysis of *Prochlorococcus* and *Synechococcus* can supply a sudden influx of organic matter, supporting increased heterotrophic growth (23, 24). While bulk chlorophyll fluorescence data in Fig. 1 and 2 reflect cyanobacteria dynamics, they do not capture heterotrophic growth, leaving it unclear whether organic matter from lysed *Prochlorococcus* cells influences heterotrophic growth in these co-cultures. Given the central role of heterotrophs in nutrient recycling and microbial community structure (4, 34, 56, 57), changes in their abundance during infection raise the possibility of indirect effects, through cross-feeding, altered MOI, or other community-level feedbacks, that could shape phage-host interactions. To examine this, we used flow cytometry to measure the abundance of both populations throughout infection and subsequent recovery in xenic *Prochlorococcus* MED4 cultures (i.e., cultures containing a heterotrophic community) infected with phage P-SSP7, allowing us to examine host-heterotroph dynamics under conditions more representative of natural communities. *Prochlorococcus* abundance initially rose for 1 day, then declined due to lysis until day 5, when growth resumed (Fig. 2d, blue line). During this lysis phase, heterotroph abundance surged—doubling more than once per day over the first 5 days (Fig. 2d, orange line). As *Prochlorococcus* began to recover, heterotroph growth slowed markedly, nearly stalling (Fig. 2d, orange line). Once *Prochlorococcus* abundance surpassed that of the heterotrophs, heterotroph growth resumed again (Fig. 2d, orange line).

To confirm the initial spike in heterotrophic growth was due to lysis, we conducted a parallel experiment comparing heterotroph abundance in xenic *Prochlorococcus* MED4 cultures with and without P-SSP7 (Fig. S2). In the no-phage control, heterotroph abundance increased after the first day, plateaued until day 4, and then increased again once *Prochlorococcus* abundance surpassed that of the heterotrophs (Fig. S2, black lines). In contrast, phage-infected cultures showed a significant increase in heterotroph abundance during the first 3 days (*t*-test *P* = <0.05 or 0.01), before leveling off at day 4, just as *Prochlorococcus* recovery began (Fig. S2b, blue line). We interpret these patterns to suggest that heterotrophs rapidly respond to organic matter released during lysis, but once this lysis-derived carbon pulse ends, their growth stops and only resumes after *Prochlorococcus* biomass increases sufficiently to enhance photosynthate supply. *Alteromonas* does not, or only minimally, alter *Prochlorococcus* growth rates under nutrient-replete conditions, and both the inorganic nutrients and residual carbon in Pro99 medium are insufficient to sustain heterotroph growth alone (32, 34). Therefore, this pattern is most consistent with organic carbon availability driving heterotroph dynamics during phage infection.

### Interaction timing between *Prochlorococcus* and heterotrophs

**Fig 3:**
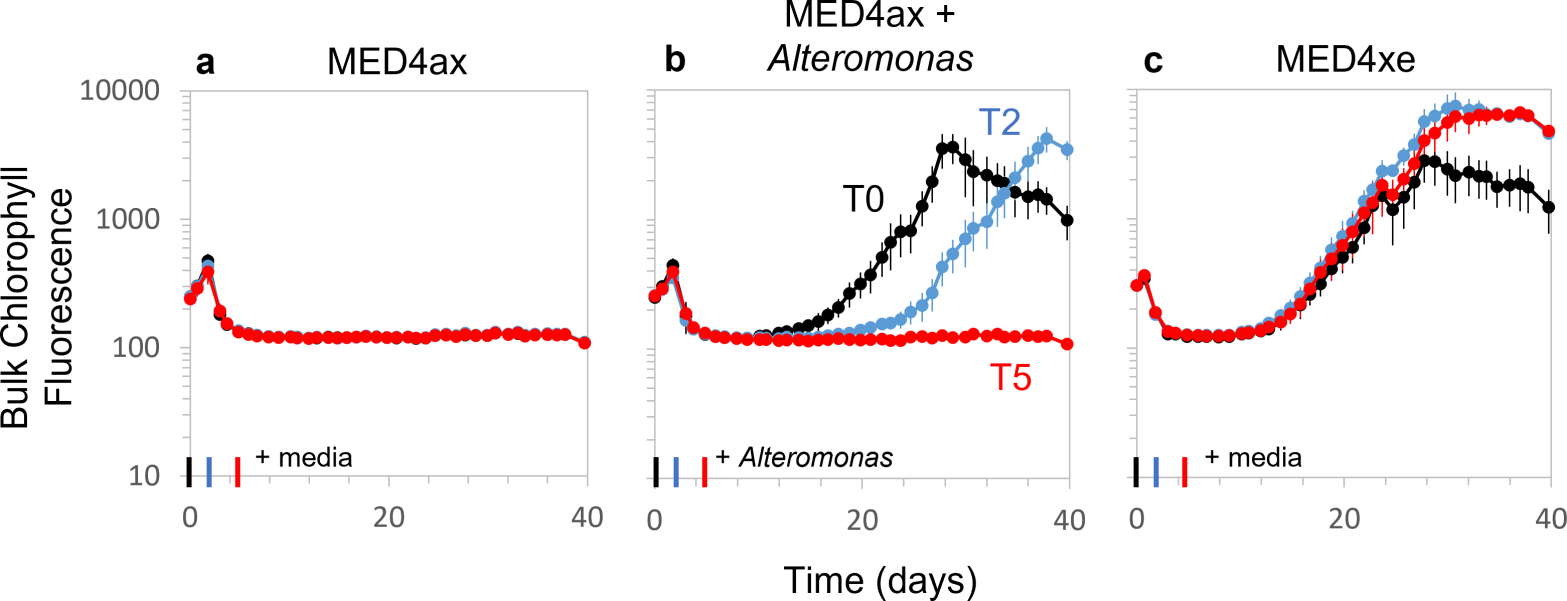
Timing of *Alteromonas* addition influences phage infection dynamics. (**a**) Axenic (“ax”), (**b**) monoxenic (+*Alteromonas*), and (**c**) xenic (community of heterotrophs) *Prochlorococcus* MED4 infected with podo-like phage P-SSP7 at the onset of the experiment. Additions of equivalent volume of media or *Alteromonas* (1 × 10^6^ cells mL^−1^) were added (as indicated by tick marks on the *x*-axis) to cultures at T0 (black), T2 (blue), and T5 (red), and the infection dynamics were monitored via bulk chlorophyll fluorescence.

Timing of recovery is not a function of phage:host ratio or initial heterotroph:host ratio. However, we observed a significant increase in heterotroph abundance during infection (Fig. 2; Fig. S2), prompting us to wonder whether this early heterotroph increase, potentially influencing host recovery through mechanisms such as cross-feeding (i.e., the exchange of metabolites between host and heterotrophic partners), plays a role. Specifically, we investigated if the timing of heterotroph introduction, including the effects of early exposure or “pre-conditioning,” would reveal how long heterotrophs must be present to affect phage lysis and recovery dynamics. Unlike previous experiments that added heterotrophs only at infection onset, we varied the timing of heterotroph addition to determine if recovery depends on early presence or if later introduction can still support it. To test this, we compared axenic and monoxenic *Prochlorococcus* MED4 cultures infected with phage P-SSP7, in which *Alteromonas* was added at specific time points: the onset of the experiment (T0), after 2 days (T2), or after 5 days (T5) (Fig. 3, black, blue, and red ticks on the axis, respectively). As a control, fresh Pro99, the same media the cells were grown in and the heterotrophs diluted into, was added to the axenic and xenic controls (Fig. 3a and c).

**Fig 4:**
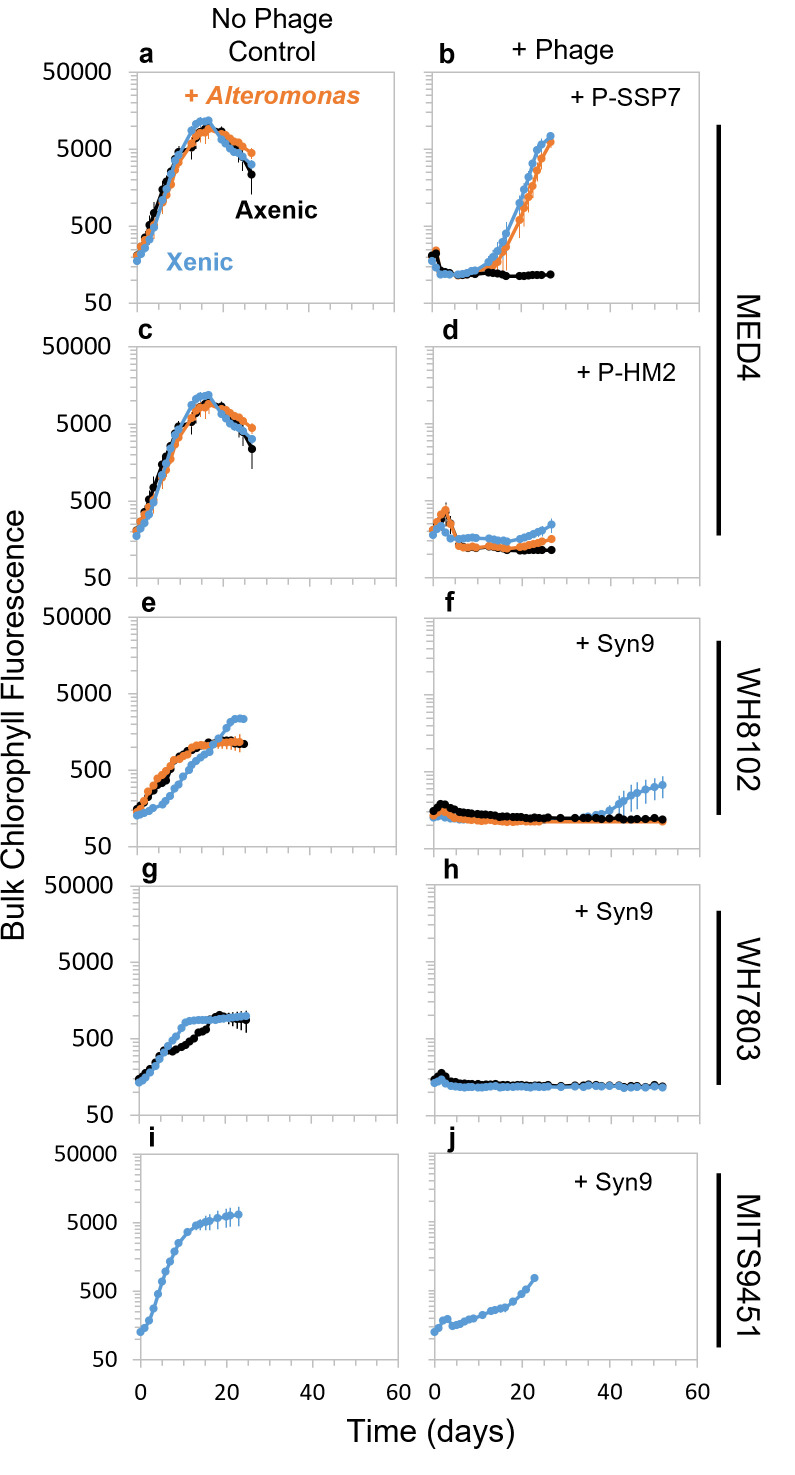
Heterotrophs facilitate recovery in multiple host-phage systems, with exceptions. Axenic (black), monoxenic (+*Alteromonas*) (orange), and xenic (community of heterotrophs) (blue) of *Prochlorococcus* MED4 without (**a and c**) or with (**b and d**) cyanophage P-SSP7 or P-HM2 or *Synechococcus* WH8102, WH7803, and MITS9451 without (**e, g, i**) or with (**f, h, j**) cyanophage Syn-9. Phages and *Alteromonas* were added at the onset of the experiment, and the infection dynamics were monitored via chlorophyll fluorescence. Xenic cultures were maintained separately from axenic cultures, each harboring an independently maintained heterotrophic community.

The timing of heterotroph addition, creating monoxenic cultures, had a significant influence on recovery time (Fig. 3b). When *Alteromonas* was added at T0, recovery proceeded as expected (Fig. 3b, black line). Addition at T2 also supported recovery, though with a noticeable delay (Fig. 3b, blue line). However, when *Alteromonas* was introduced at T5, *Prochlorococcus* failed to resume growth (Fig. 4b, red line). This suggests that in monoxenic cultures, a critical window for heterotroph-mediated population recovery exists between days 2 and 5 post-infection—possibly before the cyanobacterial population is fully lysed—and that *Prochlorococcus* must be in co-culture with heterotrophs for a minimum duration within this period to develop effective resistance or support for recovery.

To further investigate the effect of prior exposure to heterotrophs (i.e., “pre-conditioning”), we compared these outcomes with xenic cultures, which contained a natural, well-established community of heterotrophs (Fig. 3c). In xenic cultures, *Prochlorococcus* consistently resumed growth following phage infection, with recovery dynamics comparable to the T0 monoxenic treatment (Fig. 3b and c). However, the media additions, included to control for the same nutrient inputs received by the monoxenic cultures, extended the duration of exponential growth after infection, likely due to the nutrient enrichment rather than biotic interaction (Fig. 3c, blue and red lines). These results reinforce the idea that the timely presence of heterotrophs—whether already well-established in the community through preconditioning, introduced at the onset of infection, or added within a critical window—can facilitate host survival and population rebound following phage infection. Therefore, heterotrophs provide critical support to *Prochlorococcus* during phage infection, but the mechanism is unclear. To investigate this further, we examined how heterotrophs influence infection dynamics across different phage-host combinations.

### Heterotrophic influence across different host-phage systems

To begin to explore whether heterotroph-mediated recovery from phage infection is a general feature of cyanobacteria-phage interactions, we examined additional phage-host combinations. Specifically, we cultured *Prochlorococcus* MED4 and *Synechococcus* WH8102 under axenic, monoxenic, and xenic conditions. In addition, *Synechococcus* WH7803 was tested in both axenic and xenic cultures, and *Synechococcus* MITS9451 was tested in xenic cultures. Each culture was infected with either podovirus- or myovirus-like phages (P-SSP7, P-HM2, or Syn9) at the onset of the experiment. MED4 + P-SSP7 data from Fig. 1 were included for direct comparison. Control cultures without phage infection grew similarly across treatments (Fig. 4a, c, e, g, and i). Upon phage infection, all cultures exhibited lysis within 4 days, and all cultures containing heterotrophs—except for *Synechococcus* WH8102 + *Alteromonas* and xenic WH7803—resumed growth (Fig. 4b, d, f, h, and j), suggesting that heterotrophs often, but not always, facilitate recovery.

Infection outcomes varied across host-phage combinations. Regrowth of *Prochlorococcus* MED4 infected with P-HM2 was markedly slower than with P-SSP7 (Fig. 4b and d). Both P-HM2 and Syn9 are myovirus-like, with relatively long contractile tails, whereas P-SSP7 is podovirus-like with a short tail; these groups typically differ in genome size and infection dynamics (58, 59), which may contribute to the variation in host recovery observed here. Although P-HM2 produces fewer extracellular phages per infected cell, it has a shorter lytic cycle than P-SSP7 (60), which together with potential genome-encoded effects on host physiology, may result in slower host regrowth. Despite these differences, the majority of cultures containing heterotrophs (6 of 8) ultimately resumed growth.

Given the lack of recovery observed in monoxenic *Synechococcus* WH8102 cultures in Fig. 4, we wondered whether this could be due to the initial *Synechococcus*:*Alteromonas* ratio or the absence of preconditioning (i.e., prior co-culture resembling more xenic-like conditions). To test this, we compared axenic and monoxenic *Synechococcus* WH8102 cultures with treatments that varied the starting *Synechococcus:Alteromonas* ratio or included pre-conditioning with *Alteromonas* prior to the experiment. Cultures were grown with and without infection by Syn9 (Fig. S3). Consistent with earlier results (Fig. 4), none of the infected cultures resumed growth under any condition, indicating that neither the starting ratio of *Synechococcus* to *Alteromonas* nor preconditioning with heterotrophs was sufficient to support recovery after phage infection in *Synechococcus*. These results underscore that phage infection dynamics and recovery trajectories are strain- and phage-specific, highlighting that recovery post-infection is shaped by the specific microbial partners involved.

### Genetic variation in co-cultures

**Fig 5:**
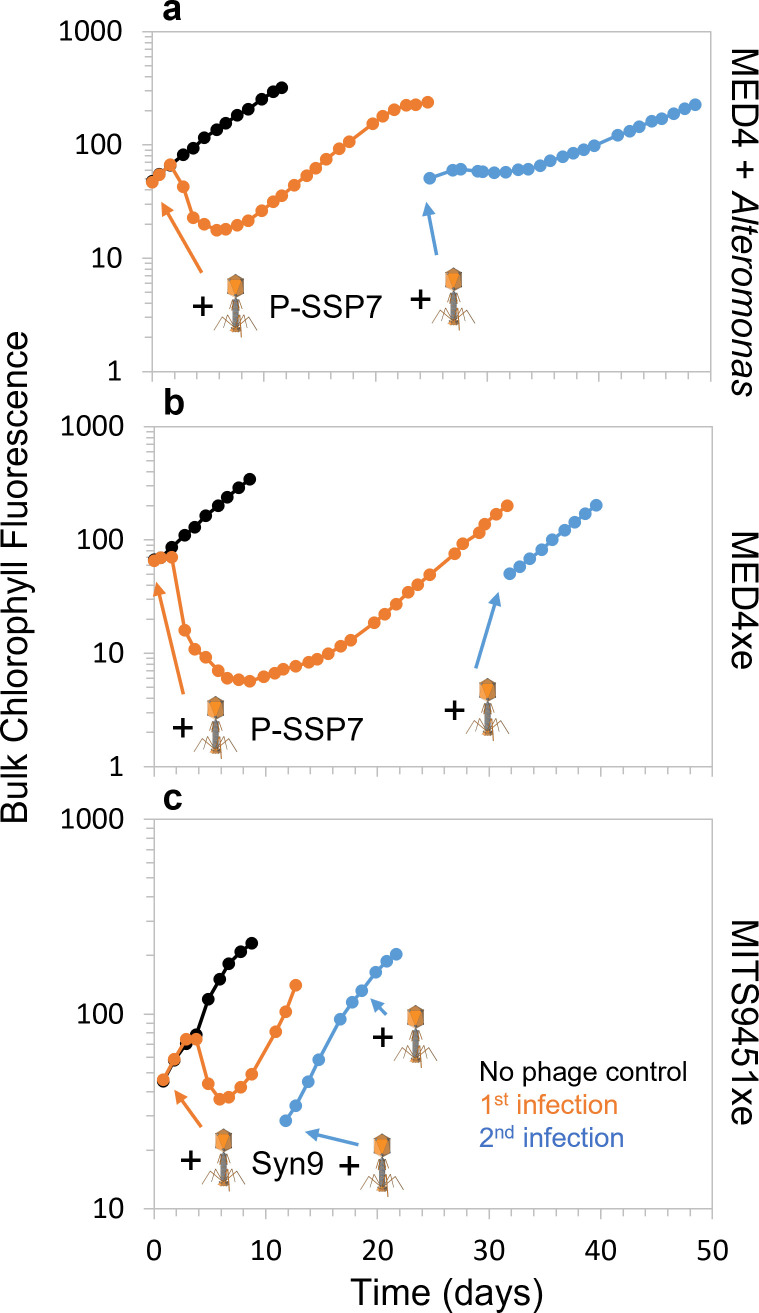
Monoxenic and xenic cultures recover from and resist consecutive phage infections. Growth of monoxenic (**a**) and xenic (**b**) *Prochlorococcus* MED4 and xenic (**c**) *Synechococcus* MITS9451 without (no-phage control, black lines) and with cyanophages (P-SSP7 and Syn9, orange and blue lines) after consecutive infections and subsequent recovery. Cultures were transferred between the first and second infections to prevent nutrient limitation. Phages were added at the onset of each experiment, with a second addition to *Synechococcus* MITS9451 in the late exponential growth phase during the second infection (blue line).

Genetic mutations conferring resistance to phage are a well-documented phenomenon in microbial systems (61, 62), including in cyanobacteria (63, 64). To explore the mechanisms driving *Prochlorococcus* and *Synechococcus* recovery when heterotrophs are present, we first investigated whether this recovery was the result of selection for phage-resistant mutants within the host population after an infection event. To test this, we grew monoxenic and xenic *Prochlorococcus* MED4 and xenic *Synechococcus* MITS9451 with and without cyanophages (P-SSP7 and Syn9, respectively) over two consecutive infection cycles, transferring recovered cells to fresh media after the first infection to assess whether resistant variants emerged (Fig. 5). All three treatments underwent a lysis phase 2–3 days into the first infection, followed by recovery (Fig. 5, orange lines). During the second infection, only the monoxenic *Prochlorococcus* culture exhibited visible lysis or growth inhibition, likely due to cells transitioning into stationary phase before the transfer, while the other treatments showed no signs of reinfection (Fig. 5, blue lines). Moreover, an additional phage dose added during the recovery phase of the second infection in *Synechococcus* cultures did not induce lysis (Fig. 5c, blue line). This pattern suggests that resistance to phage infection developed and persisted in the population after recovery, potentially reflecting the emergence of resistant phenotypes.

To investigate potential mutations driving phage tolerance, we sequenced all cultures after recovery and compared them to the no-phage controls. Mutations were considered relevant if they appeared in at least two of the three biological replicates and were either unique to controls or to post-infection samples. Notably, two variant-detection approaches (see Materials and Methods) found no evidence of genetic differences, including SNPs and indels, in the xenic *Prochlorococcus* MED4 and *Synechococcus* WH8102 post-infected cells (Table S2). In *Prochlorococcus* MED4, an in-frame 3 bp insertion resulting in the addition of a valine residue at position 100 on the protein encoded by the *prs* (ribose-phosphate pyrophosphokinase) gene—a gene involved in nucleotide biosynthesis—was observed in all but one biological replicate during the first infection and was present in all replicates following the second infection (Table S2; Fig. S4). However, this indel was also called in the no-phage control by breseq due to its lower allele frequency threshold, suggesting that it is unlikely to be a phage-driven mutation. The occurrence of this mutation in the presence of heterotrophic partners, consistent with previous observations that *prs* expression increases during co-culture with *Alteromonas* (34), further supports the idea that it reflects host-heterotroph interactions rather than phage infection. This interpretation is reinforced by the absence of mutations in xenic MED4 and MITS9451, and no lysis occurred after reintroducing phage during mid-to-late exponential growth in MITS9451 (Fig. 5). Additionally, we did not detect any larger genomic changes or additions associated with phage tolerance, such as prophages, CRISPR-Cas systems, or other mobile elements, consistent with previous reports indicating that these features are generally absent in *Prochlorococcus* and *Synechococcus* genomes (63, 65–67). We propose that the broader microbial community, through increased heterotroph abundance and interactions, such as metabolite exchange or signaling, alters host metabolism in ways that allow some cyanobacteria to survive phage infection. This community-mediated phage tolerance likely reflects phenotypic plasticity, where dynamic, reversible physiological changes—rather than genetic changes—enable host survival and prevent population collapse. However, because our analyses relied on bulk population sequencing, lineage-specific mutations arising independently within subpopulations could have escaped detection; nevertheless, the absence of consistent genetic changes across replicates supports a primarily phenotypic basis for resistance.

### Heterotroph-mediated mechanisms that shape phage tolerance in the host

We considered several heterotroph-mediated, non-genetic hypotheses, defined here as phenotypic changes that are plastic and transient, for how host populations recover from phage infection in the presence of heterotrophs. The first is non-specific adsorption of phages to non-hosts (e.g., heterotrophs), which would lower the effective infection rate (68, 69). However, under the phage-host combinations tested here, we did not observe consistent recovery in *Synechococcus* when co-cultured with one or more heterotrophs at varying host:heterotroph ratios (Fig. 4; Fig. S3). A second hypothesis concerns differences in phage infection dynamics, such as differences in adsorption kinetics, length of latent period, burst size, and extracellular decay rate, which can influence the efficiency and timing of subsequent infections (70). Yet this explanation seems unlikely, as *Synechococcus* WH8102 showed recovery in the presence of a heterotrophic community but not with a single heterotroph (Fig. 4), even though the only experimental difference was the identity of the heterotrophs.

Having ruled out these two explanations, we next consider hypotheses focused on heterotroph-mediated effects on host physiology. One possibility is that “helper” heterotrophs alleviate oxidative stress by reducing extracellular ROS through their catalase activity, a function *Prochlorococcus* cannot perform because it lacks the catalase gene, *katG* (17). By lowering environmental H_2_O_2_ concentrations, helpers could promote *Prochlorococcus* recovery and potentially reduce susceptibility to subsequent phage infections. Another hypothesis is that heterotroph-derived metabolites could provide nutritional support during stress (11, 12, 33, 56). To distinguish between these hypotheses, we tested whether *Prochlorococcus* could recover without heterotrophs by supplementing cultures with ROS scavengers, an approach particularly relevant when *Prochlorococcus*’s abundance is low and cells are more susceptible to oxidative stress (17, 18). We also considered prior findings that, under different stress conditions (e.g., prolonged darkness), *Prochlorococcus* survival is enhanced by both ROS scavengers and organic carbon supplementation (32). We therefore asked whether recovery after infection might similarly depend on heterotroph-mediated oxidative stress relief, or alternatively, whether organic carbon released by the increased abundance of heterotrophs following lysis could alter host metabolism in ways that reduce susceptibility to phage. For example, studies with *Staphylococcus* and *Escherichia coli* cells suggest that growth on different carbon sources can modulate metabolism, resulting in increased susceptibility, resistance to phage infection, or receptor expression changes (71–75).

**Fig 6:**
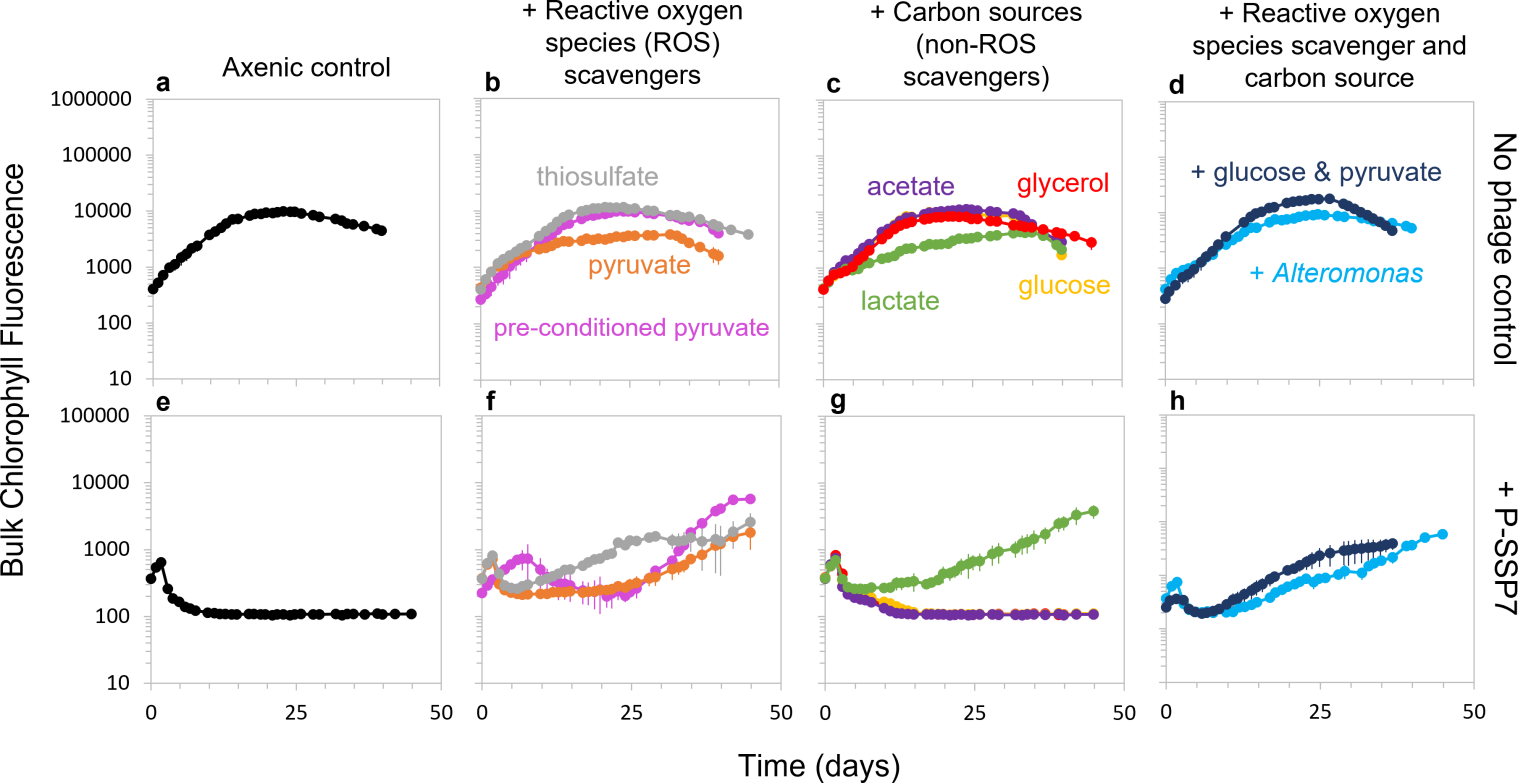
ROS mitigation and carbon supplementation enable post-infection recovery. Cultures of *Prochlorococcus* MED4 were grown alone (**a and e**; black lines), with ROS substrates (**b and f**; purple, gray, and orange lines), with carbon sources (**c and g**; green, yellow, medium blue, red lines), or with substrates or heterotrophic bacteria that can mitigate oxidative stress and provide carbon substrates (**d and h**; aqua and navy blue lines). These no-phage-control cultures were grown in parallel with cultures infected with phage P-SSP7 at the onset of the experiment. All substrates or bacteria were added at the onset of the experiment, with the exception of the cultures that were pre-conditioned with pyruvate during a previous transfer (**b and f**; purple lines).

To examine this, axenic *Prochlorococcus* MED4 cultures were treated with either inorganic (thiosulfate) or organic (pyruvate) ROS scavengers, since we aimed to test both a non-carbon and a carbon-based ROS scavenger. Additional treatments included pre-conditioned pyruvate (added to the culture in the previous transfer and at the onset of the experiment), a range of carbon sources (e.g., glucose, acetate, lactate, glycerol), and a combination of pyruvate and glucose. These were compared to untreated axenic cultures and axenic cultures supplemented with *Alteromonas*, both with and without cyanophage P-SSP7. Across the corresponding uninfected control cultures, growth rates were broadly similar among treatments, with the exception of pyruvate- and +*Alteromonas*-amended cultures, which entered stationary phase several days earlier, and lactate-amended cultures, which exhibited a slower growth rate (Fig. 6a through d). As expected, the axenic cultures failed to resume growth post-lysis (Fig. 6e). All ROS scavenger treatments supported host recovery following phage infection, with pre-conditioning with pyruvate delaying the onset of lysis and achieving a higher carrying capacity compared to other treatments (Fig. 6f). Among the tested organic carbon sources, only lactate enabled recovery, while acetate, glycerol, and glucose alone did not (Fig. 6c and g). Cultures supplemented with a combination of glucose and pyruvate had post-infection dynamics nearly identical to the axenic + *Alteromonas* treatment (Fig. 6h). Thus, pyruvate and specific carbon supplementation, particularly lactate or a combination of pyruvate and glucose, enhance *Prochlorococcus* recovery from infection with P-SSP7 by presumably mitigating ROS and supporting host metabolism.

To assess whether this trend held for different phage-host combinations, we repeated the experiment comparing axenic and monoxenic *Prochlorococcus* MED4 infected with P-HM2 and axenic and xenic *Synechococcus* WH8102 and WH7803 infected with Syn9 and compared these to axenic cultures supplemented with ROS scavenger pyruvate, organic carbon glucose, or a combination of the two. All axenic cultures infected with phage failed to recover (Fig. S5e, m, and u). In *Prochlorococcus* MED4, pyruvate alone treatment showed brief recovery signals that could not be sustained, and glucose alone resulted in a stalled population decline for ~10 days (Fig. S5f and g); however, the combination of these treatments enabled regrowth nearly identical to the +*Alteromonas* treatment (Fig. S5h). In contrast, as shown in Fig. 4 and Fig. S3, *Synechococcus* WH8102—which also lacks *katG* and may therefore be more sensitive to oxidative stress—did not recover when supplemented with pyruvate, glucose, or both (Fig. S5n through p). WH8102 failed to resume growth when co-cultured with *Alteromonas* alone (Fig. 4f), but was able to resume growth with a community of heterotrophs (Fig. S5p), suggesting that a broader suite of metabolites, supplied by diverse heterotrophs, may be necessary to alter its metabolism in a way that allows the population to resist phage infection. WH7803 did not recover when provided with any ROS scavenger or carbon sources, even when these were supplied via heterotroph-derived supplementation, suggesting that its metabolic constraints, possibly a more limited metabolic adaptability, prevent post-infection recovery (Fig. S5x).

These findings highlight that recovery dynamics differ across phage-host systems and may depend on host-specific physiological traits or interactions among microbes in the community. In *Prochlorococcu*s, ROS mitigation, particularly through pre-conditioning, and the availability of key metabolites can substitute for heterotroph-mediated recovery. The consistent success of the glucose + pyruvate combination across both phage types in *Prochlorococcus* suggests that *Alteromonas* (or other heterotrophs) may support host recovery by providing both oxidative stress relief and carbon supplementation, consistent with our findings that *Prochlorococcus* requires heterotroph-derived metabolites to survive extended darkness (12, 32, 33, 56). While *Synechococcus* WH8102 or WH7803 did not recover under similar carbon supplementation, WH8102 did recover (as well as xenic MITS9451) in the presence of a heterotrophic community (Fig. 4), indicating that such interactions can support recovery in some, but not all, *Synechococcus*-phage combinations. The recovery patterns of *Prochlorococcus* and *Synechococcus* argue against a single, universal mechanism, whether phage-mediated or simply the mitigation of oxidative stress by heterotrophs, and instead suggest that recovery depends on host-specific physiology, cross-feeding interactions with specific members of the heterotrophic community, and the host’s metabolic adaptability.

### Heterotroph composition after multiple phage infections

**Fig 7:**
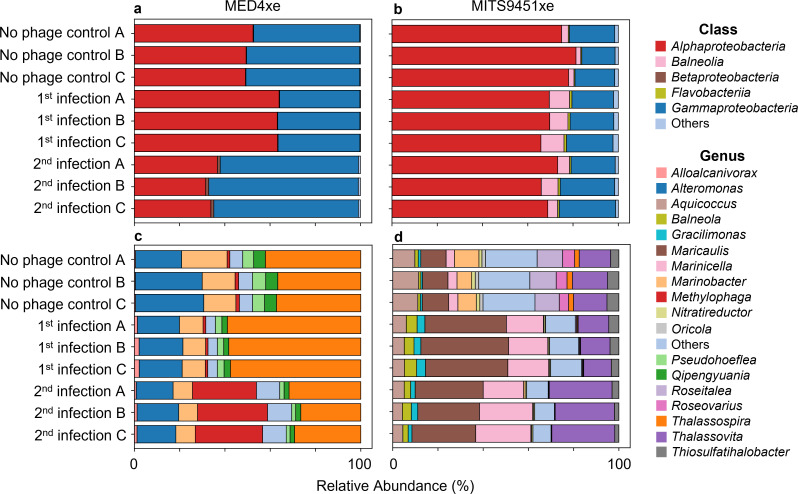
Heterotroph community composition changes following successive phage infections. Relative abundance of heterotrophic taxa as a proportion of total heterotrophic metagenomic reads was identified in xenic cultures before and after two rounds of phage infection. Data are classified to the class level for (**a**) *Prochlorococcus* MED4 and (**b**) *Synechococcus* MITS9451, and classified to the genus level for (**c**) *Prochlorococcus* MED4 and (**d**) *Synechococcus* MITS9451. Taxonomic classification was completed using ProSynTax (39) on shotgun metagenomic data generated by Illumina sequencing. Raw sequencing data are available in Table S1, and the corresponding taxonomic classification table is provided in Table S4.

Phage infection can influence host-associated microbial communities both before, during, and after lysis. Infected but intact *Synechococcus* cells release compounds capable of attracting heterotrophic bacteria (76), while lysis-associated organic matter release has also been shown to restructure heterotrophic community composition (23). These studies suggest that phage infection may actively restructure host-associated communities, although these dynamics have primarily been examined in *Synechococcus* systems. We therefore investigated whether heterotrophic community composition shifts across successive rounds of phage infection in a xenic culture of *Prochlorococcus* and compared these dynamics to those in a xenic *Synechococcus* strain not previously characterized. To assess these changes, we analyzed Illumina shotgun metagenomic sequencing data from xenic *Prochlorococcus* MED4 and *Synechococcus* MITS9451 cultures (same data from Fig. 5) for taxonomic composition, including viral abundance, using ProSynTax and its associated workflow (39) (Table S4). In all cultures, viral abundance, estimated from the proportion of metagenomic reads mapping to cyanophages, remained substantially higher than in the no-phage controls following reinfection, indicating that phages were present despite the absence of observable host lysis. We next examined whether heterotrophic community composition changed across infection time points. Both MED4 and MITS9451 cultures showed a significant effect of class and a significant class × time point interaction, where time point refers to before infection, after the first infection, and after the second infection (*F*-tests with Kenward-Roger degrees of freedom, *P <* 0.001), whereas differences across time points alone were not significant. Post hoc comparisons (Tukey contrasts with false discovery rate correction) indicated that in MED4 cultures, Gammaproteobacteria decreased and Alphaproteobacteria increased (*P <* 0.001; Fig. 7a; Table S4) following the first infection, a pattern similar to that observed in an estuarine *Synechococcus* strain (23). Betaproteobacteria and low-abundance taxa (“Others”) remained largely stable over this period. During the second attempted infection, when no host lysis occurred, Gammaproteobacteria increased, and Alphaproteobacteria decreased, while other classes remained stable. In contrast to MED4, after the first infection, MITS9451 showed a decrease in Alphaproteobacteria and an increase in Balneolia (*P <* 0.001; Fig. 7b; Table S4), highlighting host-specific differences in heterotrophic community restructuring. Even though the initial heterotrophic community compositions differed between hosts, phage infection was associated with shifts in specific classes in both systems.

At the genus level, dynamic changes largely paralleled the class-level patterns, with no overall effect of time point but a significant effect of genus and a significant time point × genus interaction for both MED4 and MITS9451 cultures (*F*-tests with Kenward-Roger degrees of freedom, *P <* 0.001), indicating taxon-specific temporal responses. Post hoc comparisons (Tukey contrasts with false discovery rate correction) showed that in MED4 cultures, *Marinobacter* (Gammaproteobacteria) and *Thalassospira* (Alphaproteobacteria) increased following the first infection, while *Methylophaga* (Gammaproteobacteria) decreased (*P <* 0.001; Fig. 7c). *Alteromonas*, which bloomed during monoxenic infections, decreased in xenic cultures, suggesting that competition in more complex communities limits its growth. During the second infection, *Methylophaga* and “Others” increased, whereas *Thalassospira* and *Pseudohoeflea* (Alphaproteobacteria) decreased (*P <* 0.05; Fig. 7c; Table S4). In MITS9451 cultures, genus-level responses were more taxon-specific but equally dynamic. Following the first infection, *Aquicoccus* (Alphaproteobacteria), *Roseitalea* (Alphaproteobacteria), *Marinobacter*, and genera in the “Others” category significantly increased, while *Balneola* (Bacteroidia), *Gracilimonas* (Bacteroidia), *Maricaulis* (Alphaproteobacteria), and *Marinicella* (Gammaproteobacteria) significantly decreased (*P <* 0.001; Fig. 7d and Table S4). *Thalassospira* and *Thalassovita* (Alphaproteobacteria) abundances displayed modest but significant changes (*P* < 0.001), whereas *Nitratireductor* (Alphaproteobacteria) and *Oricola* (Alphaproteobacteria) remained largely unchanged. During the second attempted infection, *Thalassovita* and *Maricaulis* declined, while *Thiosulfatihalobacter* (Gammaproteobacteria) increased (*P* < 0.001; Fig. 7d). Although the overall community composition differs from MED4, these results reveal similar patterns of rapid, taxon-specific shifts in response to phage infection, highlighting the dynamic restructuring of heterotrophic communities across hosts.

These results indicate that phage-mediated lysis drives rapid changes in heterotrophic community structure, likely through release of cellular contents as nutrient sources and changes in how heterotrophic populations exploit these resources. The initial increase of *Thalassospira* in MED4 cultures may reflect motility-mediated advantages in accessing phage-released metabolites (77, 78), and even in the absence of detectable host lysis, certain genera continue to shift, reflecting acclimation to changes in metabolite availability.

Overall, recovery from viral disturbance may arise from dynamic feedbacks between hosts and their associated microbial communities rather than from host-phage interactions alone, highlighting the importance of community context when evaluating the ecological consequences of viral infection.

## Data Availability

The metagenomes from this study were deposited in NCBI under BioProject PRJNA1302459. All code used to process, analyze, and generate figures from the DNA sequencing data, along with the raw data underlying all figures presented in this study, is available at https://github.com/nhinvo/pro-het-phage-interaction.

## SUPPLEMENTAL MATERIAL

Supplemental Material msystems.00769-26-s0001.pdf Tables S1 and S4; Fig. S1 to S5.

Table S2 msystems.00769-26-s0002.xlsx Sequence variants detected in xenic cyanobacterial cultures before and after phage infection (Conwill workflow).

Table S3 msystems.00769-26-s0003.xlsx Sequence variants detected in xenic cyanobacterial cultures before and after phage infection (breseq).

ASM does not own the copyrights to Supplemental Material that may be linked to, or accessed through, an article. The authors have granted ASM a non-exclusive, world-wide license to publish the Supplemental Material files. Please contact the corresponding author directly for reuse.
