# Supplementary material for: Influence of heterotrophs on phage infection of marine picocyanobacteria
Source: mSystems. 2026 Jul 20;11(8):e00769-26. doi: 10.1128/msystems.00769-26 (PMC13483391; doi:10.1128/msystems.00769-26)
Supplement: Supplementary file 1 [file msystems.00769-26-s0001.pdf]

Supplemental Material for "Influence of heterotrophs on phage infection of marine picocyanobacteria"

Table S1. Metagenomic samples before and after phage infection. Samples from monoxenic (+ *Alteromonas*) and xenic *Prochlorococcus* MED4 and xenic *Synechococcus* MITS9451 cultures collected prior to infection, after one round of phage infection, and after two rounds of phage infection, with corresponding sequencing accession numbers.

sample_name	accession	study	bioproject_accession	biosample_accession
MED4-*Alteromonas*_nophagecontrol_A	SRR34889147	SRP607052	PRJNA1302459	SAMN50479630
MED4-*Alteromonas*_nophagecontrol_B	SRR34889146	SRP607052	PRJNA1302459	SAMN50479631
MED4-*Alteromonas*_nophagecontrol_C	SRR34889135	SRP607052	PRJNA1302459	SAMN50479632
MED4-*Alteromonas*_1stinfection_A	SRR34889127	SRP607052	PRJNA1302459	SAMN50479633
MED4-*Alteromonas*_1stinfection_B	SRR34889126	SRP607052	PRJNA1302459	SAMN50479634
MED4-*Alteromonas*_1stinfection_C	SRR34889125	SRP607052	PRJNA1302459	SAMN50479635
MED4-*Alteromonas*_2ndinfection_A	SRR34889124	SRP607052	PRJNA1302459	SAMN50479636
MED4-*Alteromonas*_2ndinfection_B	SRR34889123	SRP607052	PRJNA1302459	SAMN50479637
MED4-*Alteromonas*_2ndinfection_C	SRR34889122	SRP607052	PRJNA1302459	SAMN50479638
MED4Xe_nophagecontrol_A	SRR34889121	SRP607052	PRJNA1302459	SAMN50479639
MED4Xe_nophagecontrol_B	SRR34889145	SRP607052	PRJNA1302459	SAMN50479640
MED4Xe_nophagecontrol_C	SRR34889144	SRP607052	PRJNA1302459	SAMN50479641
MED4Xe_1stinfection_A	SRR34889143	SRP607052	PRJNA1302459	SAMN50479642
MED4Xe_1stinfection_B	SRR34889142	SRP607052	PRJNA1302459	SAMN50479643
MED4Xe_1stinfection_C	SRR34889141	SRP607052	PRJNA1302459	SAMN50479644
MED4Xe_2ndinfection_A	SRR34889140	SRP607052	PRJNA1302459	SAMN50479645
MED4Xe_2ndinfection_B	SRR34889139	SRP607052	PRJNA1302459	SAMN50479646
MED4Xe_2ndinfection_C	SRR34889138	SRP607052	PRJNA1302459	SAMN50479647
MITS9451Xe_nophagecontrol_A	SRR34889137	SRP607052	PRJNA1302459	SAMN50479648
MITS9451Xe_nophagecontrol_B	SRR34889136	SRP607052	PRJNA1302459	SAMN50479649
MITS9451Xe_nophagecontrol_C	SRR34889134	SRP607052	PRJNA1302459	SAMN50479650
MITS9451Xe_1stinfection_A	SRR34889133	SRP607052	PRJNA1302459	SAMN50479651
MITS9451Xe_1stinfection_B	SRR34889132	SRP607052	PRJNA1302459	SAMN50479652
MITS9451Xe_1stinfection_C	SRR34889131	SRP607052	PRJNA1302459	SAMN50479653
MITS9451Xe_2ndinfection_A	SRR34889130	SRP607052	PRJNA1302459	SAMN50479654
MITS9451Xe_2ndinfection_B	SRR34889129	SRP607052	PRJNA1302459	SAMN50479655
MITS9451Xe_2ndinfection_C	SRR34889128	SRP607052	PRJNA1302459	SAMN50479656

Table S2. Sequence variants detected in xenic cyanobacterial cultures before and after phage infection. Single nucleotide variants (SNVs), insertions, and deletions were identified in monoxenic (+ *Alteromonas*) and xenic *Prochlorococcus* MED4 and xenic *Synechococcus* MITS9451 replicate cultures prior to infection, after one round of phage infection, and after two rounds of phage infection using the Conwill et al. workflow (1). Variants are listed with the corresponding genome position and gene ID.

Table S3. Sequence variants detected in xenic cyanobacterial cultures before and after phage infection. Single nucleotide variants (SNVs), insertions, and deletions were identified in monoxenic (+ *Alteromonas*) and xenic *Prochlorococcus* MED4 and xenic *Synechococcus* MITS9451 replicate cultures prior to infection, after one round of phage infection, and after two rounds of phage infection using breseq v0.39.0 (2) in polymorphism mode. Variants are listed with the corresponding genome position, reference nucleotide, and gene ID.

Table S4. Relative abundance of heterotrophic taxa before and after phage infection. Relative abundance of heterotrophic taxa, expressed as a proportion of total heterotrophic metagenomic reads, is shown for xenic *Prochlorococcus* MED4 and *Synechococcus* MITS9451 cultures before and after 2 rounds of phage infection. Data are presented at the community, class, and genus level based of taxonomic classification performed using ProSynTax (3) on Illumina shotgun metagenomic data.

Sample Name	Community	% Relative Abundance	# of Reads
MED4Xe_1stinfection_A	Heterotrophs	62.75105566	1634665
MED4Xe_1stinfection_A	Prochlorococcus	17.80387716	463791
MED4Xe_1stinfection_A	Unclassified	18.55247601	483292
MED4Xe_1stinfection_A	Viruses	0.892591171	23252
MED4Xe_1stinfection_B	Heterotrophs	62.64933694	1099291
MED4Xe_1stinfection_B	Prochlorococcus	19.26706572	338074
MED4Xe_1stinfection_B	Unclassified	17.25369912	302746
MED4Xe_1stinfection_B	Viruses	0.82989822	14562
MED4Xe_1stinfection_C	Heterotrophs	62.17104151	1122173
MED4Xe_1stinfection_C	Prochlorococcus	18.90522705	341235
MED4Xe_1stinfection_C	Unclassified	17.33938992	312972
MED4Xe_1stinfection_C	Viruses	1.584341518	28597
MED4Xe_2ndinfection_A	Heterotrophs	49.87309379	1428132
MED4Xe_2ndinfection_A	Prochlorococcus	23.15228187	662973
MED4Xe_2ndinfection_A	Unclassified	26.37428183	755236
MED4Xe_2ndinfection_A	Viruses	0.600342514	17191
MED4Xe_2ndinfection_B	Heterotrophs	49.76774782	4818480
MED4Xe_2ndinfection_B	Prochlorococcus	23.65974852	2290721
MED4Xe_2ndinfection_B	Unclassified	25.88005928	2505690
MED4Xe_2ndinfection_B	Viruses	0.69244437	67042
MED4Xe_2ndinfection_C	Heterotrophs	50.07253982	908404
MED4Xe_2ndinfection_C	Prochlorococcus	26.02680225	472172
MED4Xe_2ndinfection_C	Unclassified	23.43543295	425160
MED4Xe_2ndinfection_C	Viruses	0.465224984	8440
MED4Xe_nophagecontrol_A	Heterotrophs	28.23028556	541825
MED4Xe_nophagecontrol_A	Prochlorococcus	57.04989934	1094961
MED4Xe_nophagecontrol_A	Unclassified	14.71371914	282401
MED4Xe_nophagecontrol_A	Viruses	0.00609596	117
MED4Xe_nophagecontrol_B	Heterotrophs	23.70046937	435813
MED4Xe_nophagecontrol_B	Prochlorococcus	63.52216102	1168069
MED4Xe_nophagecontrol_B	Unclassified	12.77193139	234855

MED4Xe_nophagecontrol_B	Viruses	0.00543822	100
MED4Xe_nophagecontrol_C	Heterotrophs	28.05004101	507200
MED4Xe_nophagecontrol_C	Prochlorococcus	58.34049055	1054911
MED4Xe_nophagecontrol_C	Unclassified	13.60327442	245974
MED4Xe_nophagecontrol_C	Viruses	0.006194015	112
MITS9451Xe_1stinfection_A	Heterotrophs	20.18076187	522399
MITS9451Xe_1stinfection_A	Synechococcus	18.38650946	475953
MITS9451Xe_1stinfection_A	Unclassified	20.23797429	523880
MITS9451Xe_1stinfection_A	Viruses	41.19475438	1066367
MITS9451Xe_1stinfection_B	Heterotrophs	24.02501432	364779
MITS9451Xe_1stinfection_B	Synechococcus	20.1190782	305474
MITS9451Xe_1stinfection_B	Unclassified	22.06575646	335031
MITS9451Xe_1stinfection_B	Viruses	33.79015102	513046
MITS9451Xe_1stinfection_C	Heterotrophs	23.36720692	1230322
MITS9451Xe_1stinfection_C	Synechococcus	16.92313536	891031
MITS9451Xe_1stinfection_C	Unclassified	23.02812922	1212469
MITS9451Xe_1stinfection_C	Viruses	36.6815285	1931343
MITS9451Xe_2ndinfection_A	Heterotrophs	18.61098222	908893
MITS9451Xe_2ndinfection_A	Synechococcus	14.04743759	686026
MITS9451Xe_2ndinfection_A	Unclassified	18.16268937	887000
MITS9451Xe_2ndinfection_A	Viruses	49.17889082	2401719
MITS9451Xe_2ndinfection_B	Heterotrophs	21.49732249	507865
MITS9451Xe_2ndinfection_B	Synechococcus	6.006331544	141897
MITS9451Xe_2ndinfection_B	Unclassified	17.16776221	405581
MITS9451Xe_2ndinfection_B	Viruses	55.32858376	1307114
MITS9451Xe_2ndinfection_C	Heterotrophs	27.80271175	1802174
MITS9451Xe_2ndinfection_C	Synechococcus	8.111174809	525767
MITS9451Xe_2ndinfection_C	Unclassified	20.45429441	1325849
MITS9451Xe_2ndinfection_C	Viruses	43.63181903	2828218
MITS9451Xe_nophagecontrol_A	Heterotrophs	27.41222864	740114
MITS9451Xe_nophagecontrol_A	Synechococcus	48.33983409	1305147
MITS9451Xe_nophagecontrol_A	Unclassified	24.24452979	654588
MITS9451Xe_nophagecontrol_A	Viruses	0.003407482	92
MITS9451Xe_nophagecontrol_B	Heterotrophs	25.6260788	196418
MITS9451Xe_nophagecontrol_B	Synechococcus	50.66166369	388310
MITS9451Xe_nophagecontrol_B	Unclassified	23.71069191	181737
MITS9451Xe_nophagecontrol_B	Viruses	0.001565605	12
MITS9451Xe_nophagecontrol_C	Heterotrophs	26.30086569	643998
MITS9451Xe_nophagecontrol_C	Synechococcus	50.90062367	1246343
MITS9451Xe_nophagecontrol_C	Unclassified	22.79704041	558204
MITS9451Xe_nophagecontrol_C	Viruses	0.001470239	36

Sample Name	Class	% Relative Abundance	# of Reads
MED4Xe_nophagecontrol_A	Alphaproteobacteria	52.50631051	271455
MED4Xe_nophagecontrol_A	Betaproteobacteria	0.218377354	1129
MED4Xe_nophagecontrol_A	Gammaproteobacteria	46.96022205	242782
MED4Xe_nophagecontrol_A	Others	0.315090088	1629
MED4Xe_nophagecontrol_B	Alphaproteobacteria	49.42851007	204853
MED4Xe_nophagecontrol_B	Betaproteobacteria	0.19447789	806
MED4Xe_nophagecontrol_B	Gammaproteobacteria	50.0539278	207445
MED4Xe_nophagecontrol_B	Others	0.323084236	1339
MED4Xe_nophagecontrol_C	Alphaproteobacteria	49.1212936	238589
MED4Xe_nophagecontrol_C	Betaproteobacteria	0.209794241	1019
MED4Xe_nophagecontrol_C	Gammaproteobacteria	50.35514727	244582
MED4Xe_nophagecontrol_C	Others	0.31376489	1524
MED4Xe_1stinfection_A	Alphaproteobacteria	64.02577715	1033070
MED4Xe_1stinfection_A	Betaproteobacteria	0.197084391	3180
MED4Xe_1stinfection_A	Gammaproteobacteria	35.5132437	573014
MED4Xe_1stinfection_A	Others	0.263894759	4258
MED4Xe_1stinfection_B	Alphaproteobacteria	63.19325208	686031
MED4Xe_1stinfection_B	Betaproteobacteria	0.191137132	2075
MED4Xe_1stinfection_B	Gammaproteobacteria	36.36183595	394747
MED4Xe_1stinfection_B	Others	0.253774843	2755
MED4Xe_1stinfection_C	Alphaproteobacteria	63.34343119	701735
MED4Xe_1stinfection_C	Betaproteobacteria	0.191004725	2116
MED4Xe_1stinfection_C	Gammaproteobacteria	36.21218495	401168
MED4Xe_1stinfection_C	Others	0.253379141	2807
MED4Xe_2ndinfection_A	Alphaproteobacteria	36.72608536	512211
MED4Xe_2ndinfection_A	Betaproteobacteria	1.230605752	17163
MED4Xe_2ndinfection_A	Gammaproteobacteria	61.12825962	852543
MED4Xe_2ndinfection_A	Others	0.915049269	12762
MED4Xe_2ndinfection_B	Alphaproteobacteria	31.5621096	1489326
MED4Xe_2ndinfection_B	Betaproteobacteria	1.311585887	61890
MED4Xe_2ndinfection_B	Gammaproteobacteria	66.17176498	3122457
MED4Xe_2ndinfection_B	Others	0.95453953	45042
MED4Xe_2ndinfection_C	Alphaproteobacteria	33.84822149	301216
MED4Xe_2ndinfection_C	Betaproteobacteria	1.278230637	11375
MED4Xe_2ndinfection_C	Gammaproteobacteria	63.94535578	569051
MED4Xe_2ndinfection_C	Others	0.928192093	8260
MITS9451Xe_nophagecontrol_A	Alphaproteobacteria	74.93845771	502595
MITS9451Xe_nophagecontrol_A	Gammaproteobacteria	20.03348855	134360
MITS9451Xe_nophagecontrol_A	Balneolia	2.980719482	19991

MITS9451Xe_nophagecontrol_A	Flavobacteriia	0.474147764	3180
MITS9451Xe_nophagecontrol_A	Others	1.573186497	10551
MITS9451Xe_nophagecontrol_B	Alphaproteobacteria	81.3118197	144026
MITS9451Xe_nophagecontrol_B	Gammaproteobacteria	14.72212186	26077
MITS9451Xe_nophagecontrol_B	Balneolia	2.124452373	3763
MITS9451Xe_nophagecontrol_B	Flavobacteriia	0.417212411	739
MITS9451Xe_nophagecontrol_B	Others	1.424393659	2523
MITS9451Xe_nophagecontrol_C	Alphaproteobacteria	78.02996305	453809
MITS9451Xe_nophagecontrol_C	Gammaproteobacteria	17.5532985	102087
MITS9451Xe_nophagecontrol_C	Balneolia	2.385901926	13876
MITS9451Xe_nophagecontrol_C	Flavobacteriia	0.448603209	2609
MITS9451Xe_nophagecontrol_C	Others	1.582233318	9202
MITS9451Xe_1stinfection_A	Alphaproteobacteria	69.52220087	337921
MITS9451Xe_1stinfection_A	Gammaproteobacteria	18.38633755	89369
MITS9451Xe_1stinfection_A	Balneolia	8.921084142	43362
MITS9451Xe_1stinfection_A	Flavobacteriia	1.06138723	5159
MITS9451Xe_1stinfection_A	Others	2.108990211	10251
MITS9451Xe_1stinfection_B	Alphaproteobacteria	69.68377809	238830
MITS9451Xe_1stinfection_B	Gammaproteobacteria	19.29397142	66127
MITS9451Xe_1stinfection_B	Balneolia	8.071565704	27664
MITS9451Xe_1stinfection_B	Flavobacteriia	0.947673706	3248
MITS9451Xe_1stinfection_B	Others	2.003011081	6865
MITS9451Xe_1stinfection_C	Alphaproteobacteria	65.80602392	762372
MITS9451Xe_1stinfection_C	Gammaproteobacteria	20.52966127	237839
MITS9451Xe_1stinfection_C	Balneolia	10.18951864	118047
MITS9451Xe_1stinfection_C	Flavobacteriia	1.138268506	13187
MITS9451Xe_1stinfection_C	Others	2.336527655	27069
MITS9451Xe_2ndinfection_A	Alphaproteobacteria	73.20265848	618449
MITS9451Xe_2ndinfection_A	Gammaproteobacteria	19.49327983	164688
MITS9451Xe_2ndinfection_A	Balneolia	5.238002237	44253
MITS9451Xe_2ndinfection_A	Flavobacteriia	0.695748924	5878
MITS9451Xe_2ndinfection_A	Others	1.37031053	11577
MITS9451Xe_2ndinfection_B	Alphaproteobacteria	65.96834379	320337
MITS9451Xe_2ndinfection_B	Gammaproteobacteria	24.08915303	116975
MITS9451Xe_2ndinfection_B	Balneolia	7.41136592	35989
MITS9451Xe_2ndinfection_B	Flavobacteriia	0.932264123	4527
MITS9451Xe_2ndinfection_B	Others	1.598873128	7764
MITS9451Xe_2ndinfection_C	Alphaproteobacteria	68.83068705	1189322
MITS9451Xe_2ndinfection_C	Gammaproteobacteria	24.91233553	430459
MITS9451Xe_2ndinfection_C	Balneolia	4.370462326	75517
MITS9451Xe_2ndinfection_C	Flavobacteriia	0.695875617	12024

MITS9451Xe_2ndinfection_C	Others	1.190639478	20573

Sample Name	Genus	% Relative Abundance	# of Reads
MED4Xe_1stinfection_A	*Alloalcanivorax*	1.337397544	20174
MED4Xe_1stinfection_A	*Alteromonas*	18.57440608	280186
MED4Xe_1stinfection_A	*Marinobacter*	10.41597611	157120
MED4Xe_1stinfection_A	*Methylophaga*	1.212435	18289
MED4Xe_1stinfection_A	Others	4.219822706	63654
MED4Xe_1stinfection_A	*Pseudohoeflea*	2.910599741	43905
MED4Xe_1stinfection_A	*Qipengyuania*	2.388939124	36036
MED4Xe_1stinfection_A	*Thalassospira*	58.94042369	889088
MED4Xe_1stinfection_B	*Alloalcanivorax*	2.086491035	21227
MED4Xe_1stinfection_B	*Alteromonas*	19.34724786	196830
MED4Xe_1stinfection_B	*Marinobacter*	10.01804682	101919
MED4Xe_1stinfection_B	*Methylophaga*	1.058923443	10773
MED4Xe_1stinfection_B	Others	4.134057565	42058
MED4Xe_1stinfection_B	*Pseudohoeflea*	2.80374383	28524
MED4Xe_1stinfection_B	*Qipengyuania*	2.240321461	22792
MED4Xe_1stinfection_B	*Thalassospira*	58.31116799	593231
MED4Xe_1stinfection_C	*Alloalcanivorax*	2.13975469	22159
MED4Xe_1stinfection_C	*Alteromonas*	18.97244652	196476
MED4Xe_1stinfection_C	*Marinobacter*	10.24955919	106143
MED4Xe_1stinfection_C	*Methylophaga*	1.032652044	10694
MED4Xe_1stinfection_C	Others	4.227847808	43783
MED4Xe_1stinfection_C	*Pseudohoeflea*	2.995502064	31021
MED4Xe_1stinfection_C	*Qipengyuania*	2.816279865	29165
MED4Xe_1stinfection_C	*Thalassospira*	57.56595782	596145
MED4Xe_2ndinfection_A	*Alloalcanivorax*	0.810166273	10511
MED4Xe_2ndinfection_A	*Alteromonas*	16.28811119	211320
MED4Xe_2ndinfection_A	*Marinobacter*	8.553031167	110966
MED4Xe_2ndinfection_A	*Methylophaga*	28.2612449	366658
MED4Xe_2ndinfection_A	Others	10.26840082	133221
MED4Xe_2ndinfection_A	*Pseudohoeflea*	1.946834717	25258
MED4Xe_2ndinfection_A	*Qipengyuania*	2.190709333	28422
MED4Xe_2ndinfection_A	*Thalassospira*	31.6815016	411032
MED4Xe_2ndinfection_B	*Alloalcanivorax*	1.188805172	52366
MED4Xe_2ndinfection_B	*Alteromonas*	18.37517398	809413
MED4Xe_2ndinfection_B	*Marinobacter*	8.3338498	367100
MED4Xe_2ndinfection_B	*Methylophaga*	30.90752696	1361454
MED4Xe_2ndinfection_B	Others	10.68367308	470608
MED4Xe_2ndinfection_B	*Pseudohoeflea*	1.741005016	76690

MED4Xe_2ndinfection_B	*Qipengyuania*	2.283102535	100569
MED4Xe_2ndinfection_B	*Thalassospira*	26.48686346	1166727
MED4Xe_2ndinfection_C	*Alloalcanivorax*	1.161031542	9662
MED4Xe_2ndinfection_C	*Alteromonas*	17.15387453	142753
MED4Xe_2ndinfection_C	*Marinobacter*	8.565101041	71278
MED4Xe_2ndinfection_C	*Methylophaga*	29.75206413	247594
MED4Xe_2ndinfection_C	Others	10.43630609	86850
MED4Xe_2ndinfection_C	*Pseudohoeflea*	1.695043566	14106
MED4Xe_2ndinfection_C	*Qipengyuania*	1.990528617	16565
MED4Xe_2ndinfection_C	*Thalassospira*	29.24605049	243383
MED4Xe_nophagecontrol_A	*Alloalcanivorax*	0.506295383	2356
MED4Xe_nophagecontrol_A	*Alteromonas*	20.35904853	94739
MED4Xe_nophagecontrol_A	*Marinobacter*	20.10332208	93549
MED4Xe_nophagecontrol_A	*Methylophaga*	1.174622481	5466
MED4Xe_nophagecontrol_A	Others	5.696252855	26507
MED4Xe_nophagecontrol_A	*Pseudohoeflea*	4.835378787	22501
MED4Xe_nophagecontrol_A	*Qipengyuania*	5.191461745	24158
MED4Xe_nophagecontrol_A	*Thalassospira*	42.13361814	196065
MED4Xe_nophagecontrol_B	*Alloalcanivorax*	0.44615906	1685
MED4Xe_nophagecontrol_B	*Alteromonas*	29.5156063	111471
MED4Xe_nophagecontrol_B	*Marinobacter*	14.52916318	54872
MED4Xe_nophagecontrol_B	*Methylophaga*	1.541830391	5823
MED4Xe_nophagecontrol_B	Others	6.123632397	23127
MED4Xe_nophagecontrol_B	*Pseudohoeflea*	5.850906087	22097
MED4Xe_nophagecontrol_B	*Qipengyuania*	5.340669583	20170
MED4Xe_nophagecontrol_B	*Thalassospira*	36.652033	138423
MED4Xe_nophagecontrol_C	*Alloalcanivorax*	0.500107198	2216
MED4Xe_nophagecontrol_C	*Alteromonas*	30.14567653	133577
MED4Xe_nophagecontrol_C	*Marinobacter*	14.16752237	62777
MED4Xe_nophagecontrol_C	*Methylophaga*	1.569605398	6955
MED4Xe_nophagecontrol_C	Others	5.828866747	25828
MED4Xe_nophagecontrol_C	*Pseudohoeflea*	5.301000891	23489
MED4Xe_nophagecontrol_C	*Qipengyuania*	5.272565193	23363
MED4Xe_nophagecontrol_C	*Thalassospira*	37.21465567	164900
MITS9451Xe_1stinfection_A	*Aquicoccus*	6.016351933	27396
MITS9451Xe_1stinfection_A	*Balneola*	4.764372726	21695
MITS9451Xe_1stinfection_A	*Gracilimonas*	3.455515319	15735
MITS9451Xe_1stinfection_A	*Maricaulis*	36.03618244	164094
MITS9451Xe_1stinfection_A	*Marinicella*	16.40507819	74702
MITS9451Xe_1stinfection_A	*Marinobacter*	0.245520567	1118
MITS9451Xe_1stinfection_A	*Nitratireductor*	0.593817186	2704

MITS9451Xe_1stinfection_A	*Oricola*	0.082572212	376
MITS9451Xe_1stinfection_A	Others	13.1559934	59907
MITS9451Xe_1stinfection_A	*Roseitalea*	0.530350778	2415
MITS9451Xe_1stinfection_A	*Roseovarius*	0.349394653	1591
MITS9451Xe_1stinfection_A	*Thalassospira*	0.383872944	1748
MITS9451Xe_1stinfection_A	*Thalassovita*	13.51219587	61529
MITS9451Xe_1stinfection_A	*Thiosulfatihalobacter*	4.468781774	20349
MITS9451Xe_1stinfection_B	*Aquicoccus*	5.098958833	16460
MITS9451Xe_1stinfection_B	*Balneola*	4.306544696	13902
MITS9451Xe_1stinfection_B	*Gracilimonas*	3.051940609	9852
MITS9451Xe_1stinfection_B	*Maricaulis*	38.78337479	125197
MITS9451Xe_1stinfection_B	*Marinicella*	17.34606318	55995
MITS9451Xe_1stinfection_B	*Marinobacter*	0.191133512	617
MITS9451Xe_1stinfection_B	*Nitratireductor*	0.542422656	1751
MITS9451Xe_1stinfection_B	*Oricola*	0.05606996	181
MITS9451Xe_1stinfection_B	Others	12.80222793	41327
MITS9451Xe_1stinfection_B	*Roseitalea*	0.402712423	1300
MITS9451Xe_1stinfection_B	*Roseovarius*	0.339827329	1097
MITS9451Xe_1stinfection_B	*Thalassospira*	0.161704527	522
MITS9451Xe_1stinfection_B	*Thalassovita*	13.01907308	42027
MITS9451Xe_1stinfection_B	*Thiosulfatihalobacter*	3.897946476	12583
MITS9451Xe_1stinfection_C	*Aquicoccus*	5.167248645	56247
MITS9451Xe_1stinfection_C	*Balneola*	5.400499206	58786
MITS9451Xe_1stinfection_C	*Gracilimonas*	3.929155769	42770
MITS9451Xe_1stinfection_C	*Maricaulis*	36.41464766	396384
MITS9451Xe_1stinfection_C	*Marinicella*	18.07319787	196732
MITS9451Xe_1stinfection_C	*Marinobacter*	0.286900946	3123
MITS9451Xe_1stinfection_C	*Nitratireductor*	0.510321728	5555
MITS9451Xe_1stinfection_C	*Oricola*	0.054477189	593
MITS9451Xe_1stinfection_C	Others	13.72375013	149387
MITS9451Xe_1stinfection_C	*Roseitalea*	0.356260605	3878
MITS9451Xe_1stinfection_C	*Roseovarius*	0.344960952	3755
MITS9451Xe_1stinfection_C	*Thalassospira*	0.204679894	2228
MITS9451Xe_1stinfection_C	*Thalassovita*	12.19655149	132763
MITS9451Xe_1stinfection_C	*Thiosulfatihalobacter*	3.337347926	36328
MITS9451Xe_2ndinfection_A	*Aquicoccus*	5.096918524	40076
MITS9451Xe_2ndinfection_A	*Balneola*	2.833981322	22283
MITS9451Xe_2ndinfection_A	*Gracilimonas*	2.001070867	15734
MITS9451Xe_2ndinfection_A	*Maricaulis*	30.07329459	236460
MITS9451Xe_2ndinfection_A	*Marinicella*	17.83870611	140262
MITS9451Xe_2ndinfection_A	*Marinobacter*	0.707128131	5560

MITS9451Xe_2ndinfection_A	*Nitratireductor*	0.624205912	4908
MITS9451Xe_2ndinfection_A	*Oricola*	0.035356407	278
MITS9451Xe_2ndinfection_A	Others	9.537454262	74991
MITS9451Xe_2ndinfection_A	*Roseitalea*	0.230834093	1815
MITS9451Xe_2ndinfection_A	*Roseovarius*	0.254871362	2004
MITS9451Xe_2ndinfection_A	*Thalassospira*	0.010937593	86
MITS9451Xe_2ndinfection_A	*Thalassovita*	27.75020063	218194
MITS9451Xe_2ndinfection_A	*Thiosulfatihalobacter*	3.005040196	23628
MITS9451Xe_2ndinfection_B	*Aquicoccus*	4.283510599	19549
MITS9451Xe_2ndinfection_B	*Balneola*	3.945194554	18005
MITS9451Xe_2ndinfection_B	*Gracilimonas*	2.834054227	12934
MITS9451Xe_2ndinfection_B	*Maricaulis*	27.31441919	124657
MITS9451Xe_2ndinfection_B	*Marinicella*	23.51493718	107317
MITS9451Xe_2ndinfection_B	*Marinobacter*	0.335686646	1532
MITS9451Xe_2ndinfection_B	*Nitratireductor*	0.532234244	2429
MITS9451Xe_2ndinfection_B	*Oricola*	0.021473428	98
MITS9451Xe_2ndinfection_B	Others	8.739246852	39884
MITS9451Xe_2ndinfection_B	*Roseitalea*	0.124239118	567
MITS9451Xe_2ndinfection_B	*Roseovarius*	0.181647669	829
MITS9451Xe_2ndinfection_B	*Thalassospira*	0.009202898	42
MITS9451Xe_2ndinfection_B	*Thalassovita*	26.21291999	119630
MITS9451Xe_2ndinfection_B	*Thiosulfatihalobacter*	1.951233407	8905
MITS9451Xe_2ndinfection_C	*Aquicoccus*	4.492835134	72774
MITS9451Xe_2ndinfection_C	*Balneola*	2.335009899	37822
MITS9451Xe_2ndinfection_C	*Gracilimonas*	1.671030431	27067
MITS9451Xe_2ndinfection_C	*Maricaulis*	28.20310672	456828
MITS9451Xe_2ndinfection_C	*Marinicella*	24.40302041	395275
MITS9451Xe_2ndinfection_C	*Marinobacter*	0.3130674	5071
MITS9451Xe_2ndinfection_C	*Nitratireductor*	0.567299613	9189
MITS9451Xe_2ndinfection_C	*Oricola*	0.024015622	389
MITS9451Xe_2ndinfection_C	Others	8.009981609	129744
MITS9451Xe_2ndinfection_C	*Roseitalea*	0.143291153	2321
MITS9451Xe_2ndinfection_C	*Roseovarius*	0.191878028	3108
MITS9451Xe_2ndinfection_C	*Thalassospira*	0.007655365	124
MITS9451Xe_2ndinfection_C	*Thalassovita*	27.81898024	450606
MITS9451Xe_2ndinfection_C	*Thiosulfatihalobacter*	1.818828371	29461
MITS9451Xe_nophagecontrol_A	*Aquicoccus*	9.796788804	56367
MITS9451Xe_nophagecontrol_A	*Balneola*	1.476114168	8493
MITS9451Xe_nophagecontrol_A	*Gracilimonas*	1.13893514	6553
MITS9451Xe_nophagecontrol_A	*Maricaulis*	11.19868883	64433
MITS9451Xe_nophagecontrol_A	*Marinicella*	3.688286679	21221

MITS9451Xe_nophagecontrol_A	*Marinobacter*	10.83717729	62353
MITS9451Xe_nophagecontrol_A	*Nitratireductor*	1.32125514	7602
MITS9451Xe_nophagecontrol_A	*Oricola*	1.554151995	8942
MITS9451Xe_nophagecontrol_A	Others	22.84700762	131453
MITS9451Xe_nophagecontrol_A	*Roseitalea*	11.27377199	64865
MITS9451Xe_nophagecontrol_A	*Roseovarius*	5.337683059	30711
MITS9451Xe_nophagecontrol_A	*Thalassospira*	2.002043931	11519
MITS9451Xe_nophagecontrol_A	*Thalassovita*	13.90411602	79999
MITS9451Xe_nophagecontrol_A	*Thiosulfatihalobacter*	3.623979338	20851
MITS9451Xe_nophagecontrol_B	*Aquicoccus*	11.48485339	17724
MITS9451Xe_nophagecontrol_B	*Balneola*	0.970678762	1498
MITS9451Xe_nophagecontrol_B	*Gracilimonas*	0.734812895	1134
MITS9451Xe_nophagecontrol_B	*Maricaulis*	11.22890005	17329
MITS9451Xe_nophagecontrol_B	*Marinicella*	4.011663697	6191
MITS9451Xe_nophagecontrol_B	*Marinobacter*	6.485663373	10009
MITS9451Xe_nophagecontrol_B	*Nitratireductor*	1.579134942	2437
MITS9451Xe_nophagecontrol_B	*Oricola*	1.552567633	2396
MITS9451Xe_nophagecontrol_B	Others	22.58286085	34851
MITS9451Xe_nophagecontrol_B	*Roseitalea*	11.64879313	17977
MITS9451Xe_nophagecontrol_B	*Roseovarius*	4.805443058	7416
MITS9451Xe_nophagecontrol_B	*Thalassospira*	2.359306658	3641
MITS9451Xe_nophagecontrol_B	*Thalassovita*	15.55613154	24007
MITS9451Xe_nophagecontrol_B	*Thiosulfatihalobacter*	4.999190021	7715
MITS9451Xe_nophagecontrol_C	*Aquicoccus*	11.10179819	56028
MITS9451Xe_nophagecontrol_C	*Balneola*	1.080101055	5451
MITS9451Xe_nophagecontrol_C	*Gracilimonas*	0.856392728	4322
MITS9451Xe_nophagecontrol_C	*Maricaulis*	11.67384951	58915
MITS9451Xe_nophagecontrol_C	*Marinicella*	4.121464309	20800
MITS9451Xe_nophagecontrol_C	*Marinobacter*	8.141080894	41086
MITS9451Xe_nophagecontrol_C	*Nitratireductor*	1.5245455	7694
MITS9451Xe_nophagecontrol_C	*Oricola*	1.424282954	7188
MITS9451Xe_nophagecontrol_C	Others	22.97696537	115959
MITS9451Xe_nophagecontrol_C	*Roseitalea*	10.74671818	54236
MITS9451Xe_nophagecontrol_C	*Roseovarius*	4.144251251	20915
MITS9451Xe_nophagecontrol_C	*Thalassospira*	2.164165057	10922
MITS9451Xe_nophagecontrol_C	*Thalassovita*	14.79823649	74683
MITS9451Xe_nophagecontrol_C	*Thiosulfatihalobacter*	5.246148511	26476

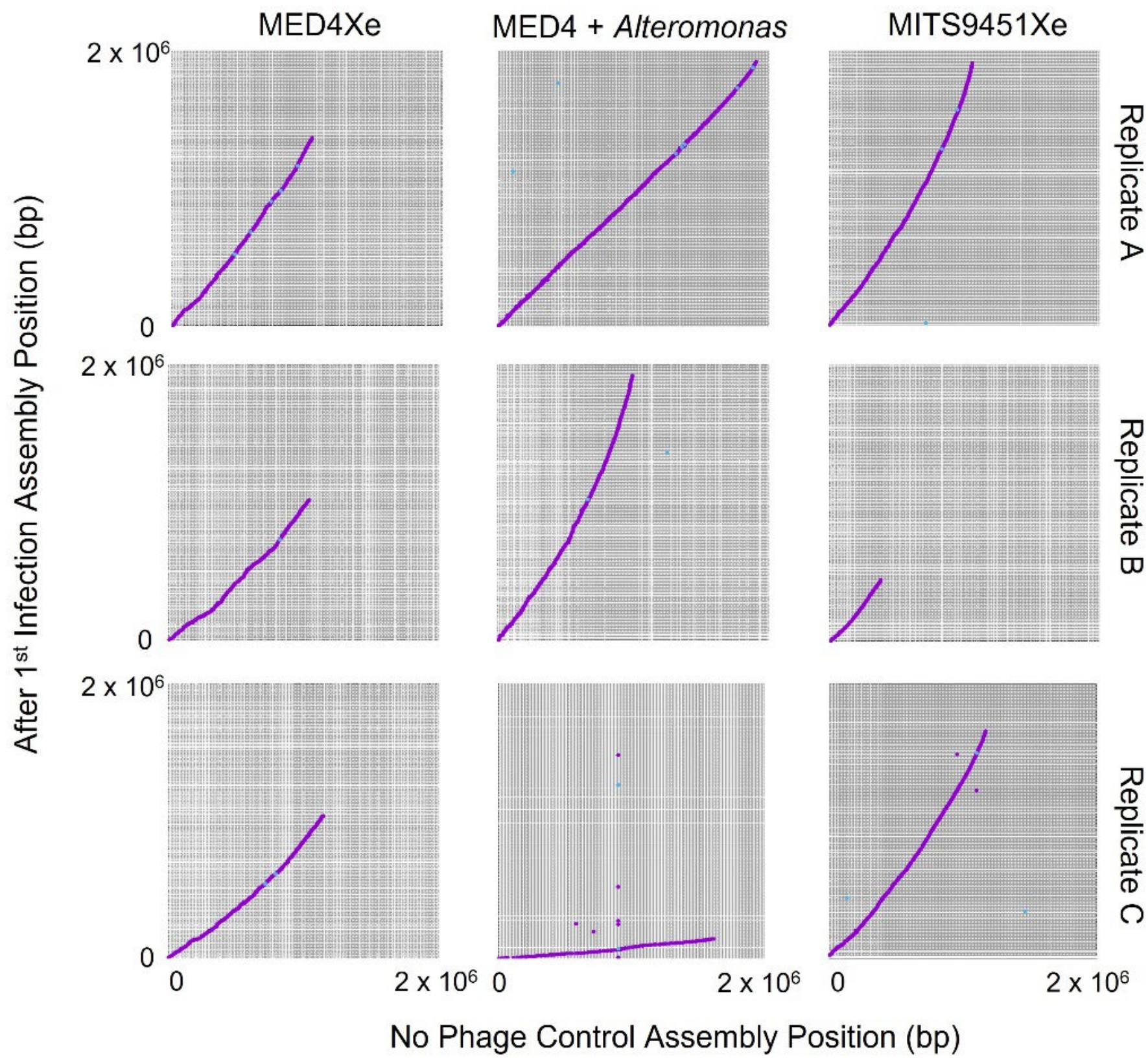

Figure S1. Cyanobacterial genomes remain stable after phage infection. De novo assembled contigs of monoxenic (+ *Alteromonas*) and xenic *Prochlorococcus* MED4 (MED4Xe) and xenic *Synechococcus* MITS9451 (MITS9451Xe) were compared before and after infection using MUMmer4. No large insertions or deletions were detected, with continuous alignments indicating genomic stability. Forward reads are shown in purple and reverse reads in blue across assembly positions (bp) for replicates A–C.

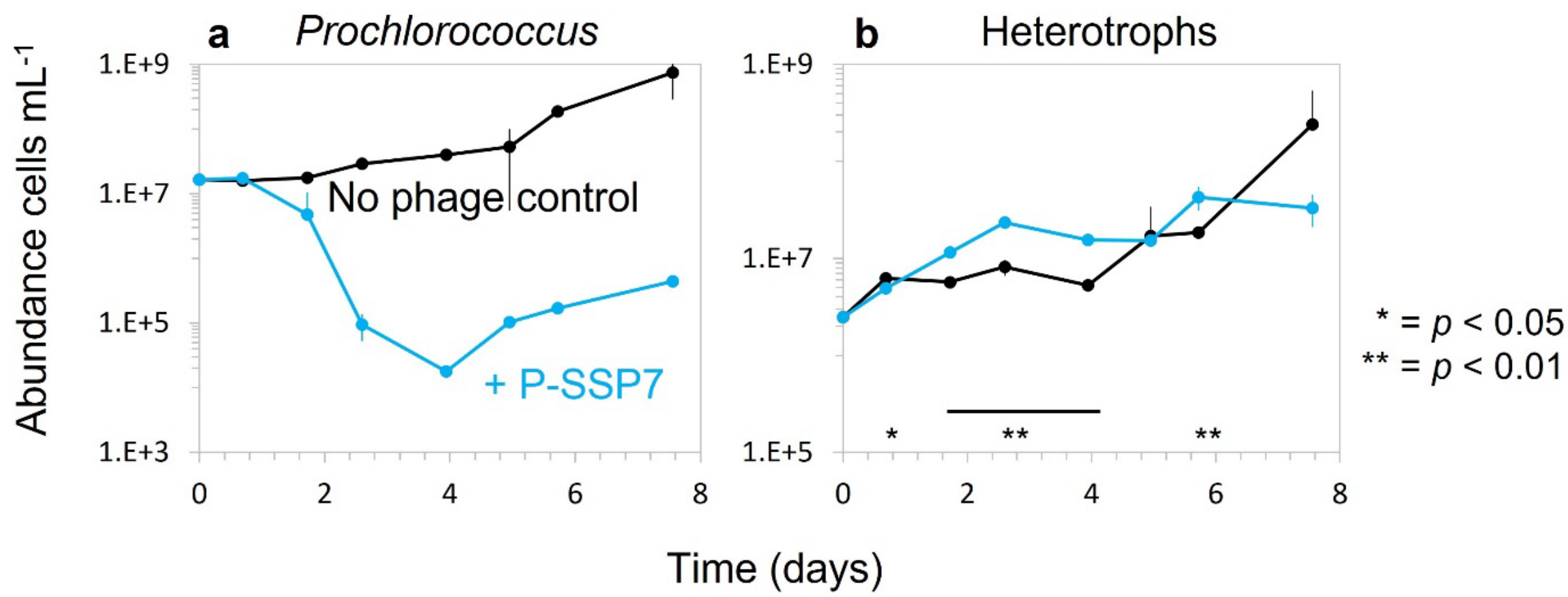

Figure S2. Heterotroph abundance increases during phage infection. Abundance of (a) xenic *Prochlorococcus* MED4 and (b) their associated community of heterotrophs with (blue) and without (black) podo-like phage P-SSP7 added at the onset of the experiment. Significance of differences in heterotrophic abundance between the no phage control and infected culture at individual time points was determined using separate *t*-tests (* = p <0.05, ** = p < 0.01), and groups of consecutive significant time points are indicated by a black line.

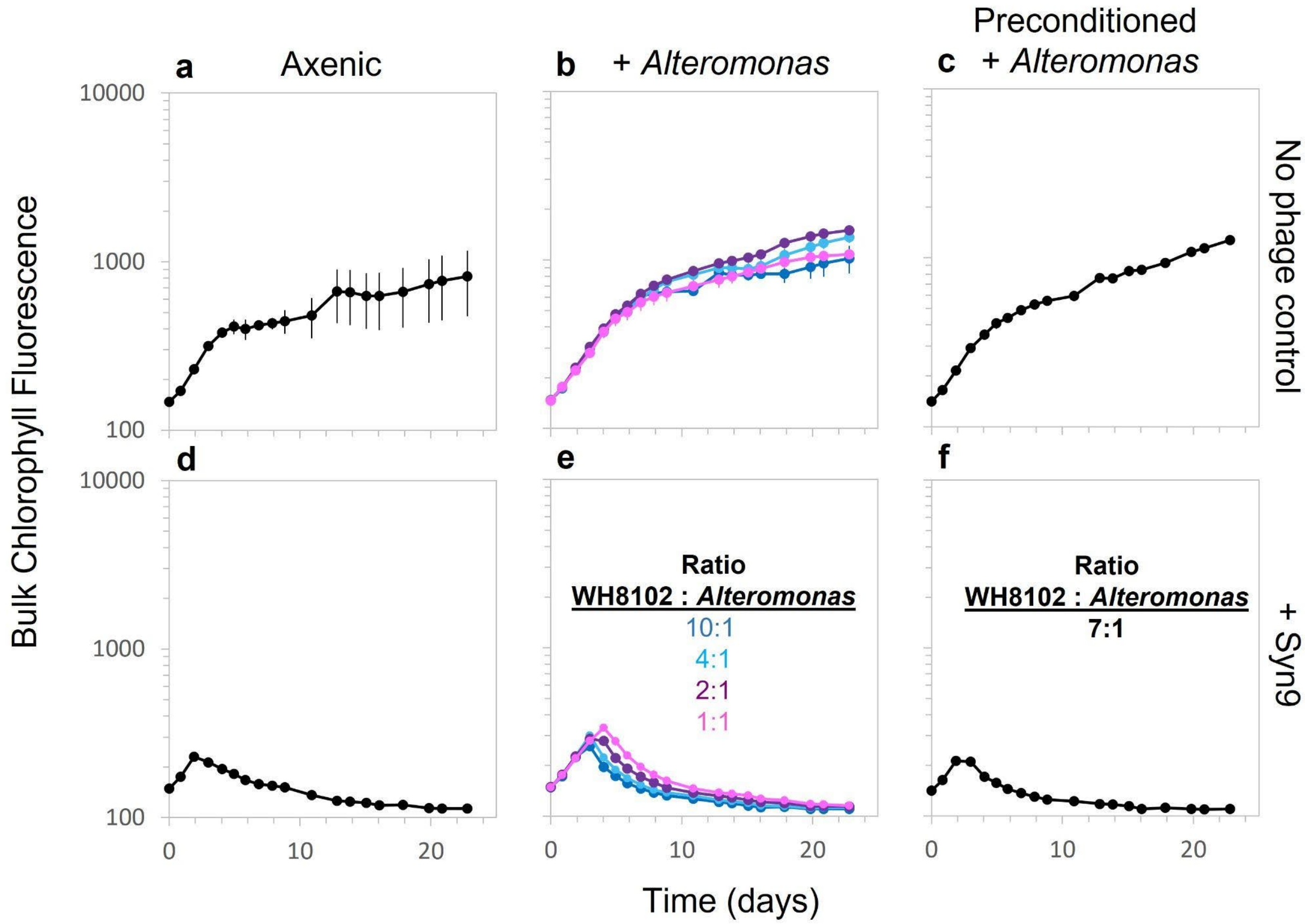

Figure S3. **Presence of *Alteromonas* does not enhance *Synechococcus* recovery post-infection**. Axenic and monoxenic (+ *Alteromonas*) with varying initial ratios of *Synechococcus* to *Alteromonas*, and preconditioned monoxenic *Synechococcus* WH8102 cultures without (a,d) and with (b, c, e, f) cyanophage Syn9 added at the onset of the experiment. Infection dynamics were monitored via chlorophyll fluorescence.

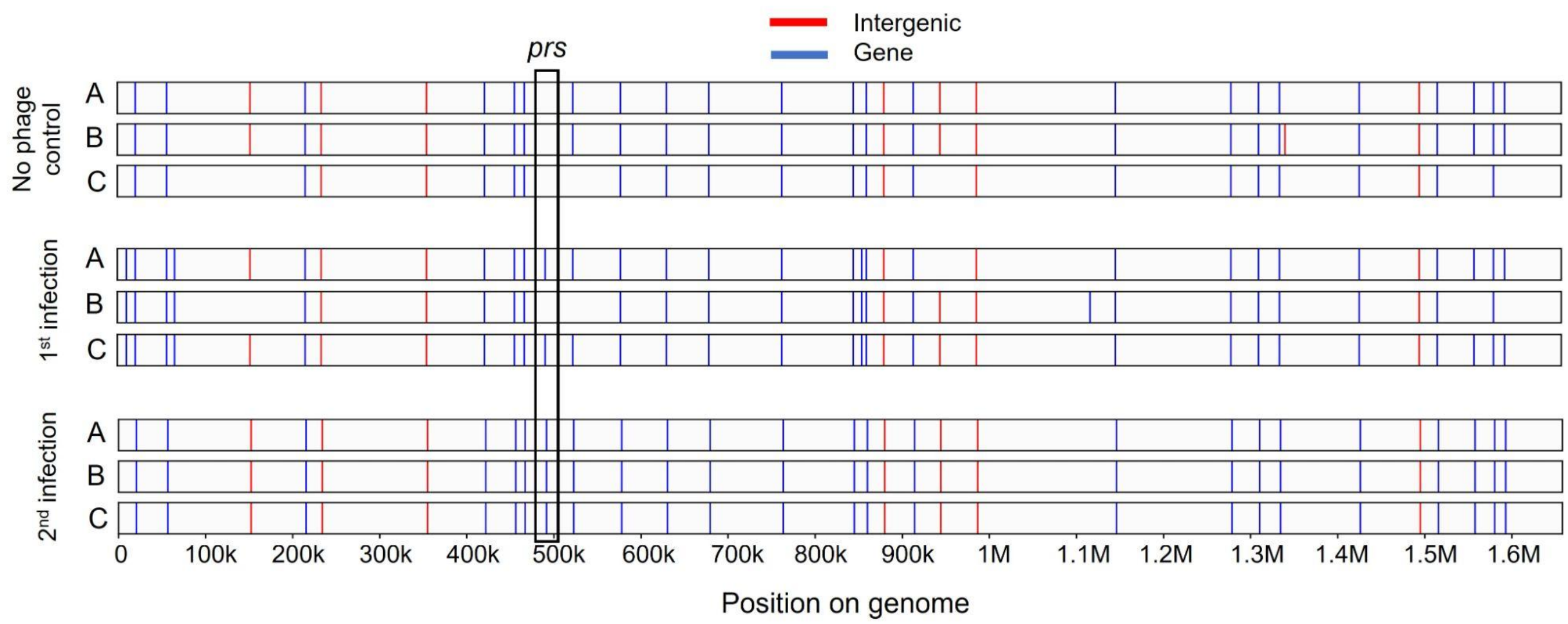

Figure S4. Single mutation found after consecutive phage infections in monoxenic *Prochlorococcus*. SNPs and indels located within genes (blue) or intergenic regions (red) are indicated across the genomes of monoxenic (+ *Alteromonas*) *Prochlorococcus* MED4 under three conditions: before infection (no phage control), after recovery from the first infection, and after recovery from a second infection using the Conwill et al. (2022) workflow (1). To be deemed significant, a mutation had to be found at least 2 of the 3 replicates relative to the control, and following both the 1st and 2nd infection. The only mutation fitting these criteria was an in-frame 3 base pair insertion in the *prs* gene, highlighted with a black box.

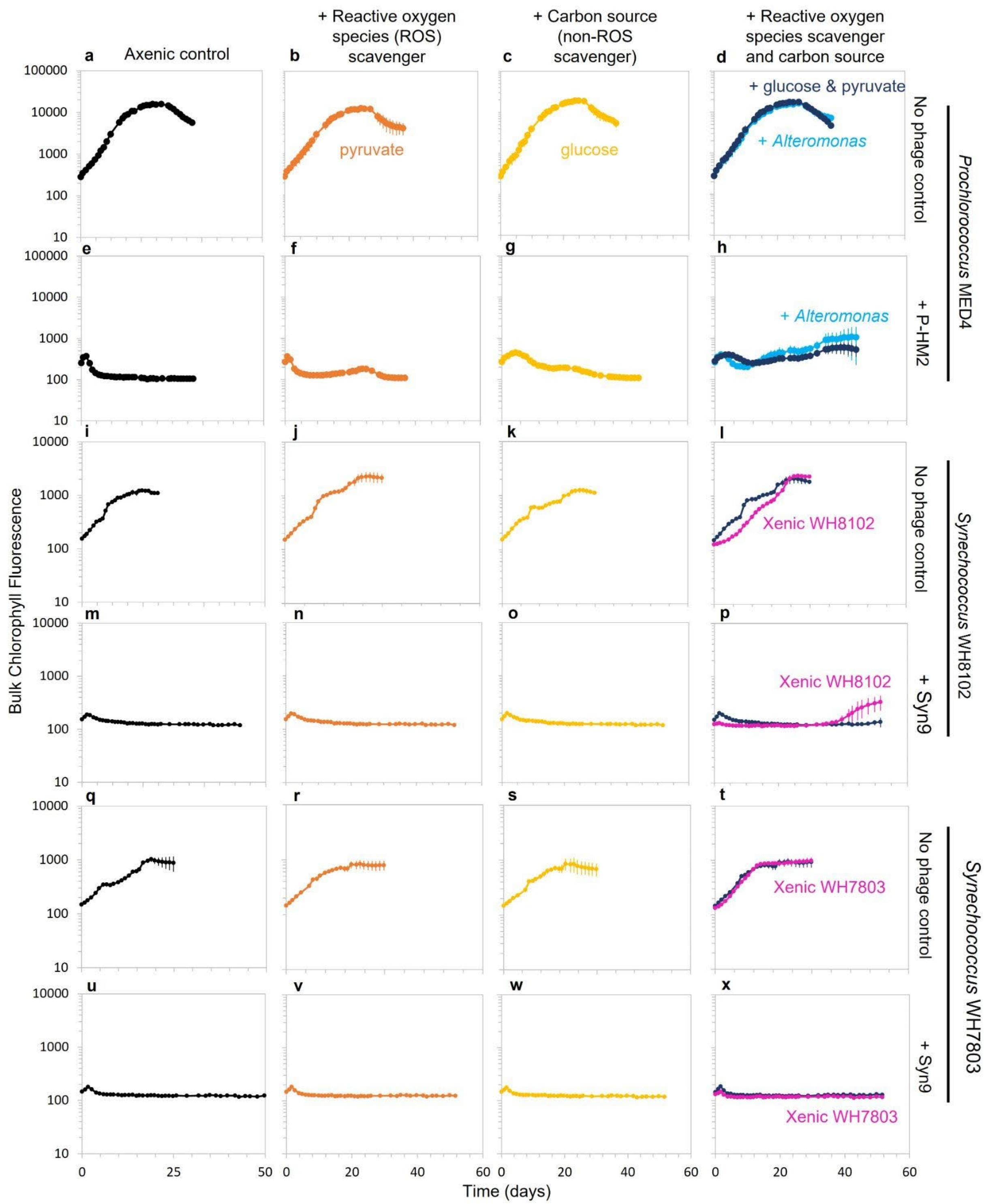

Figure S5. Reactive oxygen species (ROS) mitigation and carbon supplementation enable post-infection recovery in some phage-host combinations. Axenic cultures of *Prochlorococcus* MED4 and

Synechococcus WH8102 and WH7803 were grown alone (a, e, i, q; black lines), with reactive oxygen species scavenger (ROS) pyruvate (b, f, j, n, r, v; orange lines), with organic carbon source glucose (c, g, k, o, s, w; yellow lines), or with a combination of glucose and pyruvate or heterotrophs (either + *Alteromonas* or xenic, a community of heterotrophs) that can mitigate oxidative stress and provide carbon substrates (d, h, l, p, t, x; navy blue, aqua, or pink lines). The no phage control cultures were grown in parallel with cultures infected with either P-HM2 or Syn9 phages. All phage, substrates, or bacteria were added at the onset of the experiment, while heterotrophs in xenic cultures were already co-cultured with the host.

References

1. Conwill A, Kuan AC, Damerla R, Poret AJ, Baker JS, Tripp AD, Alm EJ, Lieberman TD. 2022. Anatomy promotes neutral coexistence of strains in the human skin microbiome. *Cell Host & Microbe* 30:171-182.e7.

2. Deatherage DE, Barrick JE. 2014. Identification of mutations in laboratory-evolved microbes from next-generation sequencing data using breseq. *Methods Mol Biol* 1151:165–188.

3. Coe A, Mullet JI, Vo NN, Anjur-Dietrich M, Berube PM, Salcedo E, Parker S, VonEmster K, Bliem C, Arellano A, Castro K, Becker J, Chisholm S. 2025. A curated protein dataset for taxonomic classification of *Prochlorococcus* and *Synechococcus* in metagenomes. *Sci Data* 12:1895.
